# Harnessing olfactory bulb oscillations to perform fully brain-based sleep-scoring and real-time monitoring of anaesthesia depth

**DOI:** 10.1371/journal.pbio.2005458

**Published:** 2018-11-08

**Authors:** Sophie Bagur, Marie Masako Lacroix, Gaëtan de Lavilléon, Julie M. Lefort, Hélène Geoffroy, Karim Benchenane

**Affiliations:** Team Memory, Oscillations and Brain States (MOBs), Plasticité du cerveau, ESPCI Paris, CNRS, PSL University, 75005 Paris, France; Universite Laval, Canada

## Abstract

Real-time tracking of vigilance states related to both sleep or anaesthesia has been a goal for over a century. However, sleep scoring cannot currently be performed with brain signals alone, despite the deep neuromodulatory transformations that accompany sleep state changes. Therefore, at heart, the operational distinction between sleep and wake is that of immobility and movement, despite numerous situations in which this one-to-one mapping fails. Here we demonstrate, using local field potential (LFP) recordings in freely moving mice, that gamma (50–70 Hz) power in the olfactory bulb (OB) allows for clear classification of sleep and wake, thus providing a brain-based criterion to distinguish these two vigilance states without relying on motor activity. Coupled with hippocampal theta activity, it allows the elaboration of a sleep scoring algorithm that relies on brain activity alone. This method reaches over 90% homology with classical methods based on muscular activity (electromyography [EMG]) and video tracking. Moreover, contrary to EMG, OB gamma power allows correct discrimination between sleep and immobility in ambiguous situations such as fear-related freezing. We use the instantaneous power of hippocampal theta oscillation and OB gamma oscillation to construct a 2D phase space that is highly robust throughout time, across individual mice and mouse strains, and under classical drug treatment. Dynamic analysis of trajectories within this space yields a novel characterisation of sleep/wake transitions: whereas waking up is a fast and direct transition that can be modelled by a ballistic trajectory, falling asleep is best described as a stochastic and gradual state change. Finally, we demonstrate that OB oscillations also allow us to track other vigilance states. Non-REM (NREM) and rapid eye movement (REM) sleep can be distinguished with high accuracy based on beta (10–15 Hz) power. More importantly, we show that depth of anaesthesia can be tracked in real time using OB gamma power. Indeed, the gamma power predicts and anticipates the motor response to stimulation both in the steady state under constant anaesthetic and dynamically during the recovery period. Altogether, this methodology opens the avenue for multi-timescale characterisation of brain states and provides an unprecedented window onto levels of vigilance.

## Author summary

Real-time tracking of vigilance states related to wake, sleep, and anaesthesia has been a goal for over a century. However identification of wakefulness and different sleep states cannot currently be performed routinely with brain signals and instead relies on motor activity. Here we demonstrate that 50–70 Hz electrical oscillations in the olfactory bulb (OB) of mice are a reliable indicator for global brain states. Recording this activity with an implanted electrode allows for clear classification of sleep and wake, without the need for motor activity monitoring. We construct a fully automatic sleep scoring algorithm that relies on brain activity alone and is robust throughout time, between animals, and after drug administration. Our method also tracks in real time the depth of anaesthesia both in the steady state under constant anaesthetic and dynamically during the recovery period from anaesthesia. Furthermore, this index predicts responsiveness to noxious stimulation under anaesthesia. Altogether, this methodology opens the avenue for characterisation of vigilance states based on OB recordings.

## Introduction

Defining the different states of brain activity and describing how they impact the computations performed by neural networks is an ongoing challenge in neuroscience. In our day-to-day lives, the most dramatic change of state is found at the frontier between sleep and wakefulness, which involves substantial modifications at the level of the brain [1–3] and throughout the whole organism [4,5]. Despite these profound transformations, to date, we surprisingly lack an easily measured marker of brain activity that allows unambiguous, moment-to-moment identification of sleep and wake states.

Sleep scoring methods in human research are now widely accepted throughout the scientific community [6,7], but there is no clear consensus for rodent studies [8]. Commonly used methods are generally completely manual or rely on manually scored training data to calibrate automatic algorithms (Table 1), therefore adding the problem of interscorer variability to the already existing issue of variability between laboratories.

**Table 1:** Overview of sleep scoring methodologies.

Reference	Motor parameters	Brain activity parameters	Manual calibration
Brankack and colleagues, 2010	EMG	EEG power: delta (1–4 Hz), theta2 (7–8.5 Hz), and gamma2 (52–70 Hz)	Manual scoring of 5% of data to train LDA or classification tree
Crisler and colleagues, 2008	EMG	EEG: 107 temporal and spectral parameters	Manual scoring of 2 h of data to train SVM
Gross and colleagues, 2009	EMG	EEG: Delta, theta, sigma, and beta bands	Manual determination of thresholds
Louis and colleagues, 2004	EMG	EEG: Delta, theta, alpha, beta, and gamma	Manual scoring of 1 d of data to establish thresholds
Rytkönen and colleagues, 2011	EMG	EEG: Delta, theta, alpha, beta, and gamma	Manual scoring of 5% of data to train naïve Bayes classifier
Veasey and colleagues, 2000	EMG	EEG: Delta, sigma, theta	Manual determination of thresholds
Zeng and colleagues, 2012	Doppler and video recording of movement and breathing	none	Manual scoring of 10% data to train SVM
Liang and colleagues, 2012	EMG	13 EEG features	Manual scoring of 2 animals to establish thresholds for all others
Stephenson and colleagues, 2009	EMG	EEG: delta, alpha, theta, beta, and gamma power	No manual steps

Abbreviations: EEG, electroencephalography; EMG, electromyography; LDA, linear discriminant analysis; SVM, support vector machine.

Moreover, all current sleep scoring methods essentially rely on motor activity to discriminate sleep from wake (see Table 1). This renders all methods inherently vulnerable to any mismatch between these brain states and the level of motor activity such as during freezing—a commonly used behaviour in mice—or any sleep anomalies causing movement during sleep [9].

The state of the art therefore presents both a conceptual and a technical problem regarding the identification of sleep and wake.

Sleep scoring methods are based on the assumption that the information about sleep states is contained in classically recorded signals and that the challenge is to extract the correct marker from them [10]. In order to implement a reliable sleep scoring, the candidate marker of sleep and wake must show a systematic and sustained difference in value throughout each state.

Despite multiple studies, such a clear-cut situation has never been found when using brain signals [11]. Therefore, attempts to identify sleep with brain signals rely on more elaborate methods that extract composite features from local field potential (LFP) data [12]. However, the first step of dimensionality reduction is highly dependent on the data at hand and often results in mapping data onto axes that vary between animals and sessions, requiring post hoc human labelling procedures [12]. To compensate for the poor quality of the brain-related sleep markers, machine learning has been used in several studies but with no decisive improvement [13–16].

Here we propose a novel brain-related marker allowing us to reliably track transitions from sleep to wakefulness: gamma power (50–70 Hz) measured in the olfactory bulb (OB). This oscillation has been previously shown to vary between sleep states [17], but we show here that it displays the desired characteristics to continuously identify these states.

This oscillation is strongly suppressed during sleep and continuously present during waking. Importantly, the distribution of gamma power follows an easily separable bimodal distribution, the optimal situation for an automatic separation procedure. Coupling this indicator with the classical hippocampal theta/delta power ratio allows us to construct a fully automated sleep scoring algorithm that classifies wake, rapid eye movement (REM) sleep, and non-REM (NREM) sleep based on brain state alone.

We then use these variables to construct a robust 2D phase space that is highly robust across mice—from the same and different strains—and days. This phase space forms the basis of an innovative analytical methodology for the study of fast timescale transitions using kinematic modelling of trajectories. As an application, we provide strong evidence for a deep asymmetry between transitions from sleep to wake and wake to sleep. In particular, the awakening transition displays strongly driven, fast dynamics, whereas the process of falling asleep is more stochastic and slower.

Finally, we show that OB LFP activity is a remarkably versatile indicator of global brain states: beta power can be used to discriminate REM and NREM sleep, while gamma power not only tracks changes in vigilance between sleep and wake but also predicts in real time the depth of anaesthesia.

## Results

### OB gamma power modulation throughout brain states

**Fig 1:**
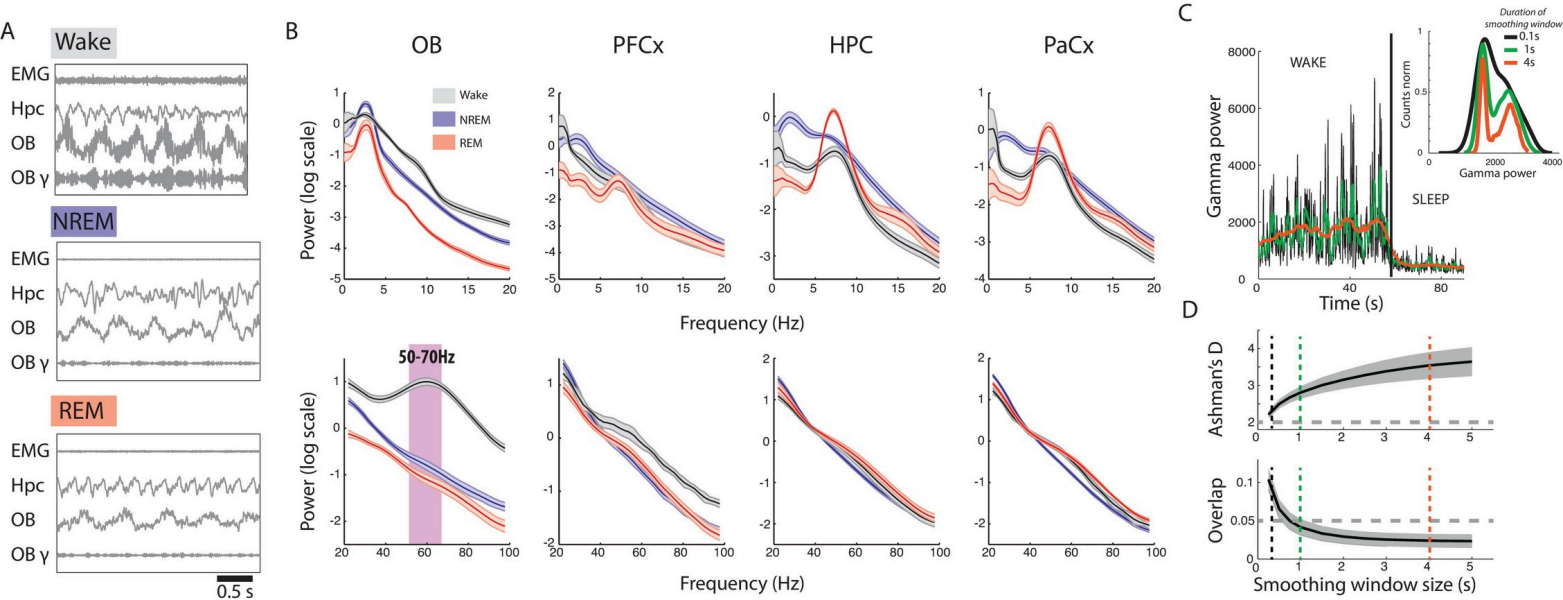
Identification of OB gamma as a reliable marker of sleep/wake states. (A) EMG, HPC, and OB activity during wake, NREM, and REM sleep. Wake is characterised by high EMG activity and high gamma power in the OB, whereas sleep is characterised by low EMG activity and low gamma power in the OB. HPC LFP shows regular theta activity during REM sleep. Filtered signals from the OB in the gamma band (OB-γ, 50–70 Hz) illustrate the remarkable decrease in gamma power during sleep states. (B) Low-frequency (top) and high-frequency (bottom) spectra from different brain regions during NREM, REM, and wake states as classified using movement-based scoring (EMG or filmed activity). Note the strong increase in gamma activity in the OB in the wake state (pink bar). (*n* = 9 for OB and HPC, *n* = 6 for PFCx and PaCx, error bars: s.e.m.). Note that differences in HPC activity between vigilance states vary strongly according to recording depth; see S5 Fig. (C) Gamma power in OB drops as the animal transitions from wake to sleep. The data are smoothed with different window lengths (0.1, 1, and 4 s, as indicated by colour). Inset: histogram of gamma values after different smoothing windows have been applied. The fast fluctuations present in the awake state are smoothed out as the window size increases, yielding a more clearly bimodal distribution with a larger smoothing window. (D) Ashman’s D (bimodality index, significant if larger than 2) for gamma power increases (top), and the overlap between the two Gaussians fit to the bimodal distribution of gamma power decreases (bottom) with the length of the smoothing window. The stars show window sizes illustrated in C. EMG, electromyography; HPC, hippocampus; NREM, non-REM; OB, olfactory bulb; PaCx, parietal cortex; PFCx, prefrontal cortex; REM, rapid eye movement.

Classical sleep scoring methods differentiate sleep from wake states using electromyography (EMG) activity or the animal’s motion recorded using accelerometers or video tracking (see Table 1, [11,14,15,18–23]). Theta and delta power recorded in the hippocampus (HPC) or cortex (due to the volume conduction of theta oscillations) can then be used to discriminate REM from NREM sleep. According to classical sleep scoring methods, wake is defined by high EMG activity and irregular HPC activity during quiet wake or theta oscillations during active exploration. Sleep, in contrast, is defined by low EMG power. NREM is discriminated from REM sleep using the theta/delta power ratio in the HPC: during NREM, delta power is strong, whereas highly regular theta oscillations are observed during REM (Fig 1A). Similar results can be obtained when recording electroencephalography (EEG) rather than LFP.

In order to construct a sleep scoring method that relies on brain signals alone, we screened multiple brain regions to find a good predictor for discriminating between sleep and waking states. We recorded from multiple brain regions in 15 freely moving mice: the OB, the HPC, the prefrontal cortex, and the parietal cortex. Mice were recorded for an average of 6.6 ± 0.58 h (minimal recording length: 2 h) in their home cages in the light period and slept on average 58% of the time. We initially used a classical sleep scoring method based on movement and hippocampal activity to establish a database of recordings from different brain states using 10 mice that were also implanted with an EMG wire in the nuchal muscles (*n* = 6) or tracked using video (*n* = 4).

The average spectra over wake, NREM, and REM periods are shown in Fig 1B. In cortical and hippocampal areas, as expected, REM and NREM showed strong differences in the theta and delta band, and wake periods showed less low-frequency power. In cortical and hippocampal areas, no individual frequency band allowed a clear discrimination between sleep and wake.

However, we found a strong increase in power in the OB during waking relative to sleep states. This difference was strongest in the low-gamma band centred at 60 Hz, as previously described [17]. OB activity is modulated by the breathing cycle in all vigilance states but displays a sustained gamma band oscillation only in the wake state (Fig 1A). Crucially, gamma power was low in both NREM and REM sleep states and could therefore allow the distinction of wake from REM sleep. Gamma activity is therefore a candidate replacement for muscular activity in sleep scoring.

OB gamma power displays strong fluctuations correlated with breathing activity on the scale of around a second [17]. To find the appropriate timescale for tracking the changes in gamma power related to brain state changes, we applied a smoothing window of varying length to the instantaneous gamma power. As the smoothing window increased in length, the distribution of gamma power became more distinctly bimodal and the two underlying distributions clearly separated (Fig 1C and 1D). We found that smoothing windows of 1 s or above produced two normal distributions that overlapped by less than 5% (Fig 1D bottom). This analysis allowed us to establish a set of parameters (frequency, smoothing window) for which gamma power in the OB is a promising marker for discriminating between wake and sleep on fine timescales of the order of 1 s without any reliance on muscular activity.

### Construction and validation of the sleep scoring algorithm

**Fig 2:**
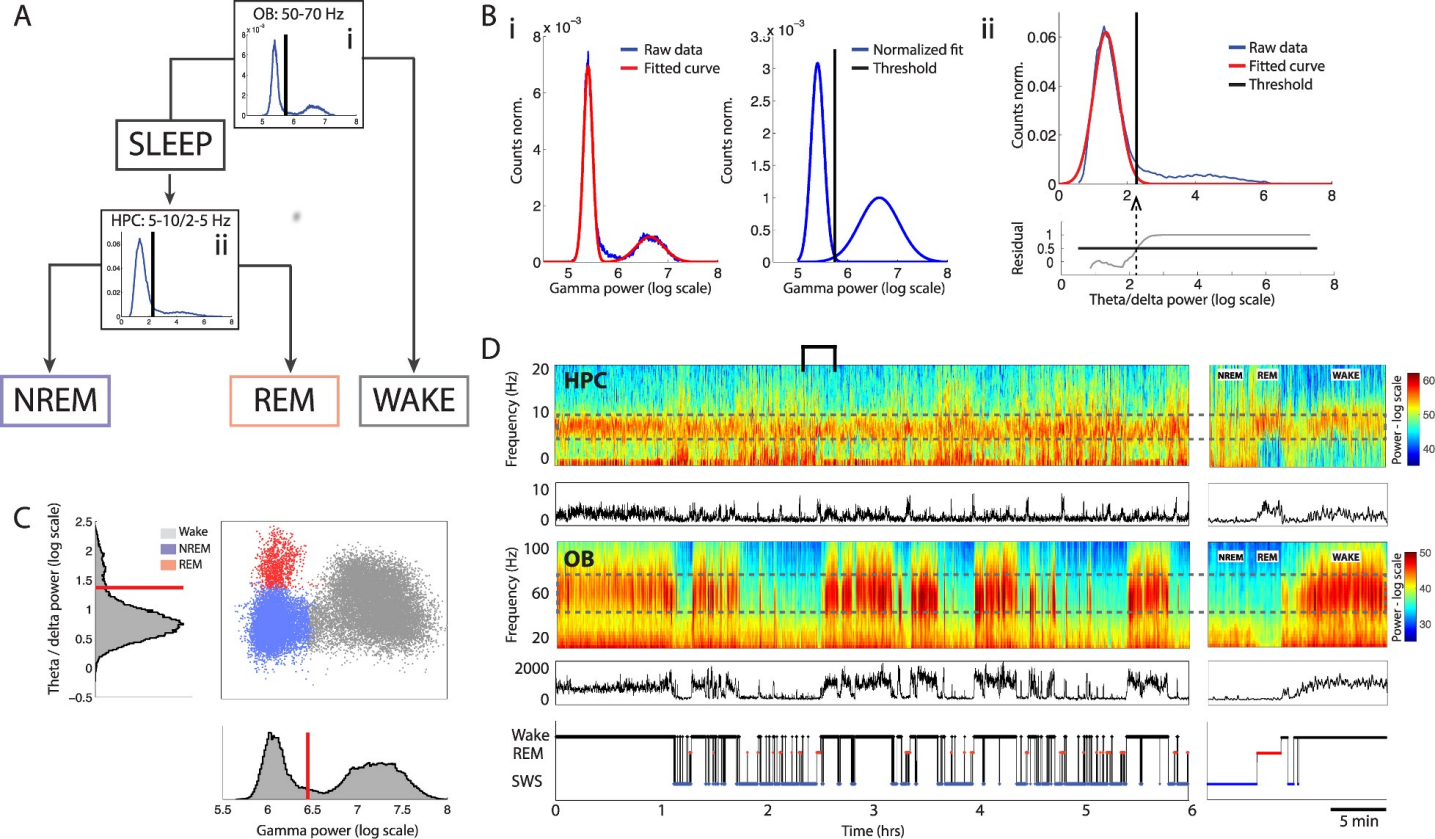
Schematic of sleep scoring method and application to example data set. (A) Flowchart of data through the scoring algorithm. Sleep and wake states are first classified based on OB gamma (i). Sleep data are then further classified into REM and NREM sleep based on HPC theta/delta power ratio (ii). (B) Example of automatic thresholding of distributions. (i) Two Gaussian distributions are fit to the distribution of OB gamma power (left), and their areas are equalised (right). The threshold is placed at the intersection of the two distributions. (ii) A Gaussian distribution is fit to the distribution of HPC theta/delta power ratio during sleep. The residuals are shown in the bottom plot. The threshold is placed at the point where the fit explains less than 50% of the data. (C) Phase space of brain states showing the distribution of NREM (blue), REM (red), and wake (grey) data for an example mouse. Corresponding histograms are shown along the relevant axis, with automatically determined thresholds in red. (D) HPC low-frequency spectrogram with theta/delta power ratio below, OB high-frequency spectrogram with gamma power below, and hypnogram for the same mouse as in C. The relevant frequency bands are outlined by a dotted grey line. Right: bracketed area on an expanded timescale. HPC, hippocampus; NREM, non-REM; OB, olfactory bulb; REM, rapid eye movement.

A schematic of the sleep scoring algorithm is shown in Fig 2A. All steps are automatic and do not require any supervision by the user.

The first step classifies data into sleep and wake periods. Instantaneous smoothed gamma power in the OB shows a bimodal distribution that can be well fit by a sum of two Gaussian functions (Fig 2B) (mean R^2^ = 0.98 ± 0.009). The two component distributions correspond to gamma power during sleep and wake periods, respectively. Since the amplitude of these distributions depends on the proportion of time spent in each state, they are normalised to unit area, and the sleep/wake threshold is defined as the intersection of the two corresponding Gaussian curves (Fig 2Bi). Below-threshold values of gamma power are defined as sleep and above-threshold values as wake.

The second step classifies sleep data into REM and NREM periods. The theta-to-delta ratio in the HPC during sleep shows a clear peak of low values corresponding to the predominant NREM phase and a flat shoulder of higher values corresponding to the rare REM phase (Fig 2Bii). Fitting a Gaussian distribution to the lower values allows us to automatically place a threshold to discriminate REM from NREM sleep.

Each time point is now attributed to one of the three states based on its OB gamma power and HPC theta/delta ratio. Short epochs of any give state lasting less than 3 s are merged with the neighbouring states. An example session is shown in Fig 2C and 2D that illustrates the construction of a 2D phase space for brain states (Fig 2C). This space demonstrates the clear separation of brain states even after the merging and dropping of short epochs. This shows that the continuity hypothesis does not lead to any aberrant classification (see S1 Movie for data from another mouse).

**Fig 3:**
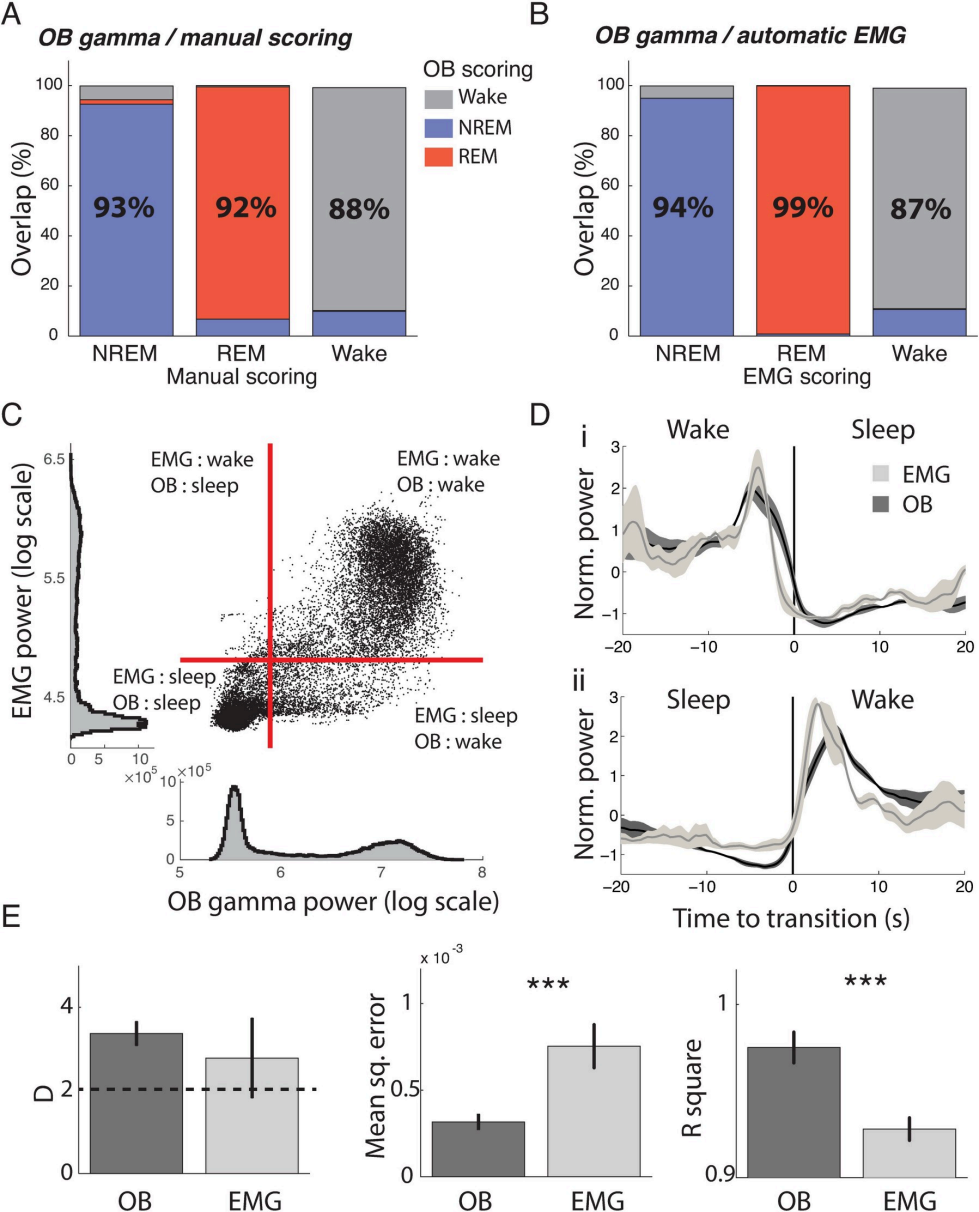
Validation of new sleep scoring method by comparison with classical methods. (A) Overlap of manual scoring with OB gamma scoring. Each column gives the percent of time from each state identified using manual scoring (x label) that is classified as NREM (blue), REM (red), and wake (grey), respectively, using OB gamma scoring. For example, the first column can be read as signifying that out of all the data classified as NREM by the manual scorer, 93% was also classified as NREM by the OB gamma scoring, 2% as REM, and 5% as wake (*n* = 4). (B) Overlap of automatic EMG scoring with OB gamma scoring (*n* = 6). (C) Correlation of OB gamma power and EMG power for one mouse, with automatically determined thresholds in red. Dots in the upper right and lower left-hand corner are identically classified by both approaches. (D) OB gamma and EMG power triggered on transitions from wake to sleep and sleep to wake determined by the OB gamma, demonstrating tight temporal locking of changes with both indicators (*n* = 6). (E) Quality of fit of two Gaussians to OB gamma power and EMG power distributions. Both distributions are strongly bimodal, as the average Ashman’s D is larger than 2. However, both mean square error and R^2^ show that gamma power distributions are better fit by a sum of two Gaussians (*n* = 15 for gamma, *n* = 6 for EMG; *t* test, *p* = 0.43, 0.0063, 0.0005, respectively, *** *p* < 0.001). EMG, electromyography; NREM, non-REM; OB, olfactory bulb; REM, rapid eye movement.

We validated the sleep scoring algorithm by comparing it to manual sleep scoring performed using HPC LFPs and EMG activity, the classical golden standard. Two expert scorers independently scored sessions from 4 mice with an average interscorer overlap of 89% ± 3% (Cohen’s κ: 0.81). On average, the OB-based and manual sleep scoring overlapped by 90% ± 2% (Cohen’s κ: 0.83) throughout the sessions (Fig 3A). We also performed scoring using an automatic EMG algorithm. Agreement between the OB gamma scoring and the automatic EMG scoring was 93% (Cohen’s κ: 0.85) (Fig 3B). Moreover, EMG power and OB gamma power were highly correlated and time locked at transition points (Fig 3C and 3D). Some time points show disagreement between the two variables (Fig 3C); this shows that by coupling OB gamma activity and EMG measurements, we can hope to clarify the definition of quiet wake and movement during sleep.

We next compared how clearly bimodal the distributions of these two variables were to evaluate how well they separated sleep and wake states. Both variables were strongly bimodal, according to Ashman’s D (Fig 3E, left); however, the error made when fitting their distributions with two Gaussians was higher for EMG power (Fig 3E, centre and right). We found that this higher error was explained by a larger proportion of values in the trough between the two Gaussians for EMG power (11% ± 2% for EMG and 4% ± 3% for gamma power). This indicates that ambiguous intermediary values between clearly defined sleep and wake are more frequent when using EMG than OB LPF, leading to more potential errors in scoring.

This demonstrates that sleep scoring using gamma power in the OB or using EMG, with either automatic or manual methods, gives very similar classification of brain states, confirming that OB gamma power is a good predictor of wake and sleep as classically defined. Moreover, gamma power provides distributions with a clearer separation than EMG power, making it a more reliable predictor.

### Robustness of the sleep scoring method

**Fig 4:**
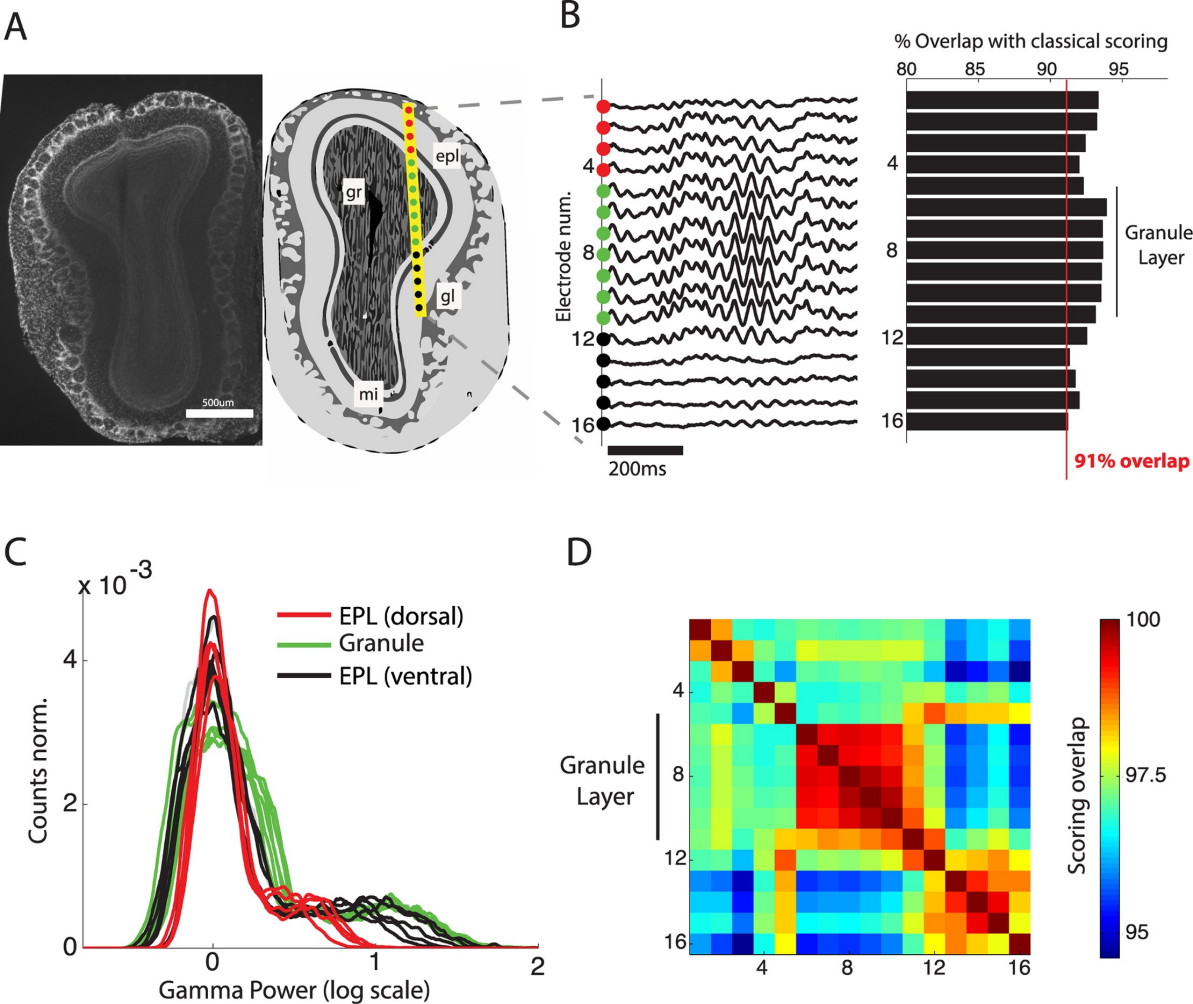
Robustness of sleep scoring method to variations in implantation site. (A) Anatomical position (right) of 16-site silicon probe in the OB as estimated from histology (left). Sites 1–4 and sites 12–16 are respectively dorsal and ventral to the gl in the epl. (B) Sleep scoring is performed using the gamma activity from each electrode site and compared to sleep scoring using the animal’s movement. Accuracy is calculated as total overlap in sleep/wake periods. (C) Gamma power distributions for each electrode classified by implantation location: dorsal epl, gl, and ventral epl. Note that the strong separation of sleep and wake peaks is present in all layers and is clearest for electrodes within and below the granule layer. (D) Correlation matrix of sleep scoring performed using gamma power from different implantation depths. All values are high (above 95%), and the gl shows particularly coherent scoring (>99%). epl, external plexiform layer; gl, glomerular layer; gr, granule cell layer; mi, mitral cell layer; OB, olfactory bulb.

Sleep scoring is often performed on large batches of animals, requiring simple surgeries and a high success rate. It is known that theta rhythm can be easily recorded in the hippocampal area using a single LFP wire. How does the OB gamma power used for sleep scoring depend on the exact placement of the recording site? To answer this question, we simultaneously recorded activity from multiple depths in the OB covering the outer and inner plexiform layers, the mitral cell layer, and granular cell layer using a 16-site linear probe (Fig 4A). We found that gamma oscillations could be observed at all depths, and sleep scoring performed using electrodes at all depths highly overlapped (>92%) with classical, movement-based sleep scoring (Fig 4B). We however observed that the separation between wake and sleep peaks was best in the deeper recording sites, and in particular, the most coherent scoring was found in those sites within the granule cell layer where gamma oscillations are visibly stronger (Fig 4C and 4D).

This demonstrates that placement of the LFP wire for reliable scoring does not require great precision during implantation, assuring good scoring for all implanted animals. The granule cell layer, however, appears to be the optimal anatomical region to ensure reliable scoring, since it shows the clearest bimodality of gamma power. The coordinates we recommend aim for the centre of this zone (anteroposterior [AP] +4, mediolateral [ML] +0.5, dorsoventral [DV] −1.5). We also observed that gamma power recorded in the piriform cortex could be used for reliable scoring (S4 Fig).

**Fig 5:**
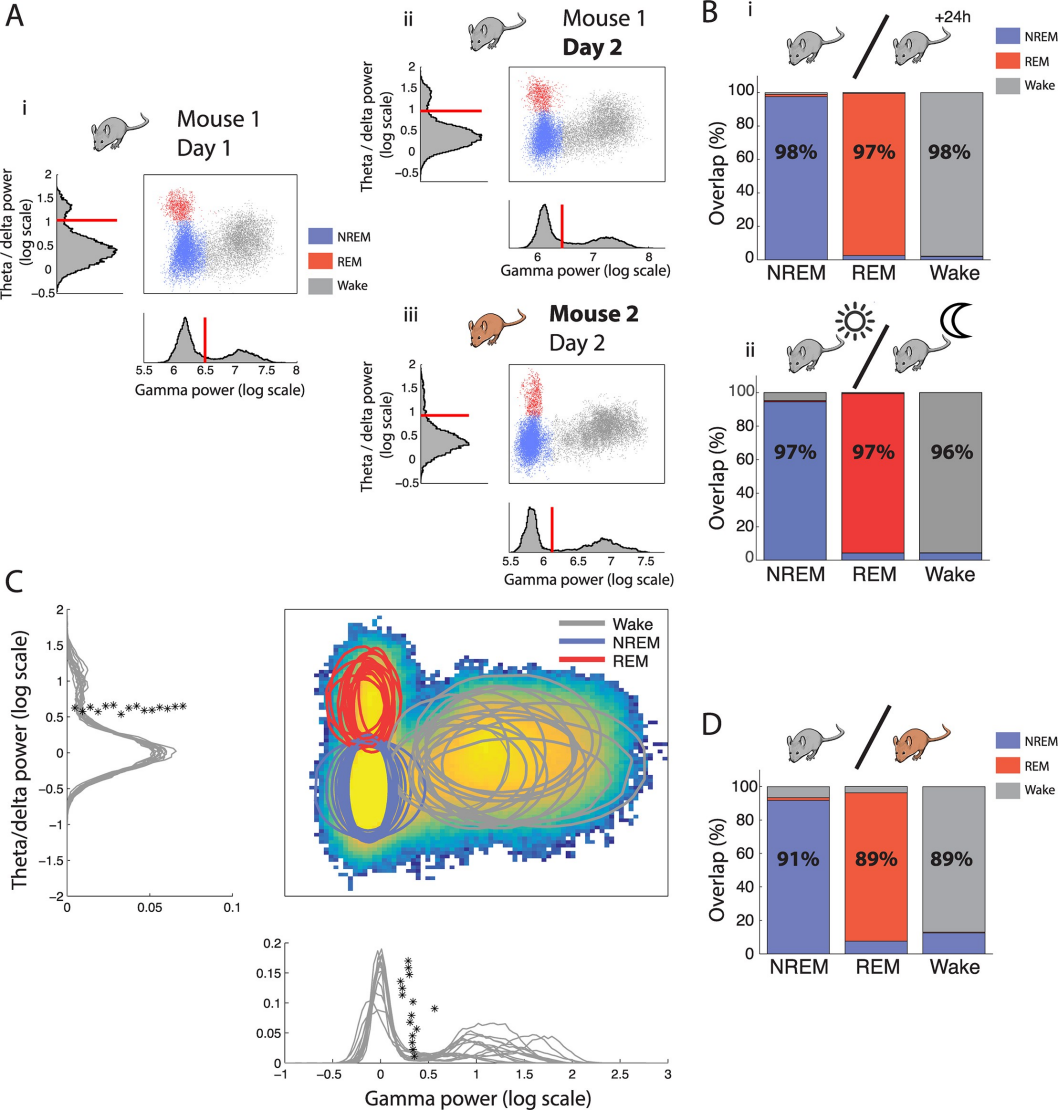
Robustness of sleep scoring method throughout time and between animals. (A) Phase space of brain states and corresponding histograms along the relevant axis with automatically determined thresholds of the same mouse over different days (i versus ii) and in two different mice (i versus iii) demonstrating the highly conserved architecture across time and individuals. (B) Overlap of scoring using thresholds determined on the same mouse on one day and applied the following day (i) and during successive light and dark periods (ii). Each column gives the percent of time from each state identified using thresholds determined on the reference day data that are classified as NREM (blue), REM (red), and wake (grey), respectively, using thresholds determined using data from different days (i) or during the light period (ii) (*n* = 10 mice were recorded on consecutive days and used for interday scoring, *n* = 5 mice were recorded over a 24 h period of light and dark periods). (C) Heat map of point density averaged over all phase spaces for all mice (*n* = 15). Circles show the 95% boundaries of NREM, REM, and wake for each mouse. Histograms from all mice are shown along the relevant axis with automatically determined thresholds (*). The data from each mouse are normalised by dividing the instantaneous power by the average NREM power to align all distributions to the peak of distribution of NREM activity. (D) Overlap of scoring using thresholds determined on one mouse and applied to a different mouse (*n* = 15 mice). As in B. NREM, non-REM; REM, rapid eye movement.

An optimal sleep scoring technique must provide reproducible results in the same animal throughout time and easily comparable results between animals. In other words, the phase space used to define sleep states must be stable. This phase space was constructed so that the separation between wake and sleep on the one hand and REM and NREM on the other hand used orthogonal axis. This simple space is remarkably consistent among animals and across days, as can be seen by the similar position of the clouds of points representing each state (Fig 5A).

We first quantified this similarity in the same animals between days and between light and dark cycles. We used the thresholds defined for one animal on a given light cycle to score test data from the same animal on a subsequent light or dark cycle. The scoring was then compared with that obtained using thresholds determined on the test data.

We found that the observed consistency between phase spaces was sufficient to perform highly accurate scoring on the next day’s light cycle (average over recordings: 97% ± 0.5%, Cohen’s κ: 0.97, *n* = 15, Fig 5Bi) and during the following dark cycle (average over recordings: 96% ± 0.9%, Cohen’s κ: 0.94, *n* = 4, Fig 5Bii) using independently defined thresholds. We also found that stable recordings could be obtained for up to 10 wk with maintained thresholds (S1 Fig). Finally, since gamma oscillations in the OB have been linked with information processing and novelty [24], we exposed 8 mice to a novel environment for 15 min, during which the animals actively explored. On average, only 2% ± 1.1% of the time was misclassified as sleep. This demonstrates that any changes in gamma activity linked to behaviour remain well within the bounds of the wake state as previously defined.

We next asked whether our method would allow for easy comparison between animals by comparing the phase space used for sleep scoring across individuals. We found that after normalising distributions to the mean NREM values, to account for changes in implantation depth between animals, both OB and HPC distributions were highly reproducible across mice, and the independently determined thresholds had very close values (Fig 5C). Scoring one animal using the thresholds determined for another, we found that scoring was also highly reliable (average over recordings: 90% ± 2.5%, Cohen’s κ: 0.85, *n* = 15, Fig 5D).

We tested whether our novel algorithm was applicable to studies using drug administration by applying it to mice injected with the classical antidepressant fluoxetine that is well known for its REM-suppressing effect. Sleep scoring using this method reproduced the well-known reduction in REM sleep as expected, but more importantly, it agreed with classical EMG-based scoring after drug delivery, and no change in the phase space was observed (S2 Fig).

We also verified that the method was applicable in three other mouse strains: Gad2-IRES-Cre knock-in C57BL/6J Rj (*n* = 4), C3H/HeNRj (*n* = 2), and DBA/2Rj (*n* = 3) (S3 Fig). Blindly applying the algorithm to these strains produced remarkably similar phase space of brain states and a good overlap with classical EMG methods in all lines. Indeed, despite some large changes in the OB power spectra between strains, they all shared the common feature of a difference between sleep and wake in the 50–70 Hz band, which allowed us to generalise our methodology. For C3H and more particularly for DBA mice, the OB spectrum appears as a smooth 1/f slope; however, subtraction of the spectra from sleep and wake states (S3B Fig) clearly reveals the wake-specific 50–70 Hz oscillation that is otherwise obscured by the other oscillation at 30 Hz. It is only by using multiple mice strains recorded during different brain states that we can clearly identify that the apparently continuous OB spectrum in these strains can be decomposed into two different peaks.

This demonstrates that brain state–related changes in OB gamma power are quantitatively robust over multiple days and throughout the circadian cycle. Moreover, the phase space thus constructed is highly reproducible between animals. Finally, the method can be applied to studies involving pharmacological manipulation and generalised to other mouse strains. This makes OB gamma power an excellent parameter to use for automatic methods of scoring and a promising tool for comparing sleep in cohorts of animals.

### A powerful tool to study mismatch between brain state and motor activity

A major issue with current approaches to sleep scoring is that EMG activity conflates absence of movement and sleep, which suffers from notable exceptions such as freezing behaviour. Freezing is a widely studied behaviour in paradigms such as fear conditioning. It is defined as a complete absence of all movement except for respiration. This absence of movement is associated with a strong drop in EMG power. Although it has been shown that on average EMG power is lower during sleep than freezing [25], we investigated whether freezing could be misclassified as sleep using EMG power and whether OB gamma power could resolve this issue. Six mice were therefore fear conditioned by pairing tones with mild foot shocks and, during test sessions, displayed robust freezing to tone presentation (post conditioned stimulus not associated with shock [CS−] freezing: 25% ± 10%; post conditioned stimulus associated with shock [CS+] freezing: 67% ± 13%).

**Fig 6:**
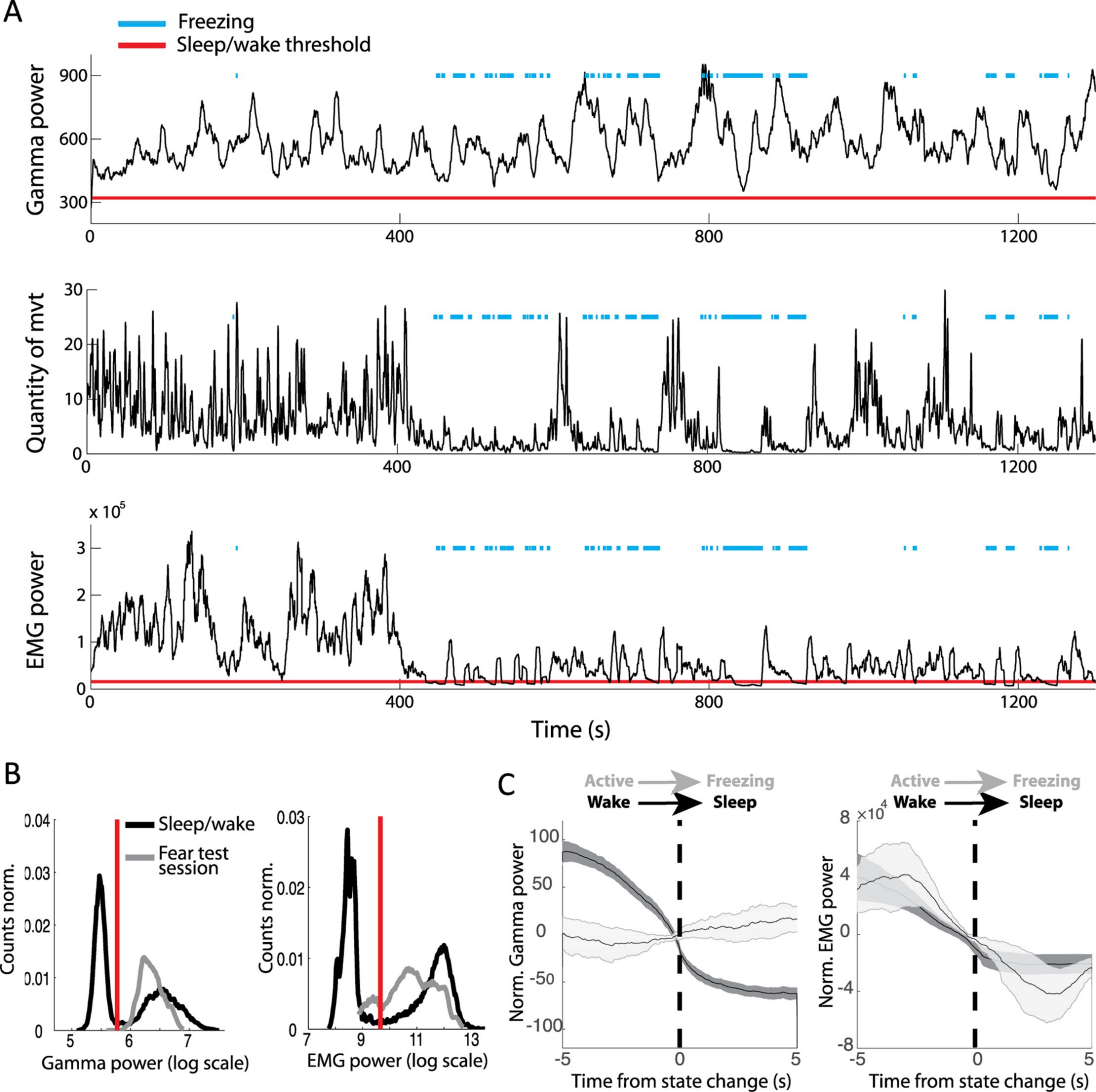
Correct classification of freezing periods as wake using OB gamma power but not EMG power. (A) OB gamma power, quantity of movement, and EMG power during an example test session during which the mouse displayed freezing episodes (blue line). Red lines indicate the sleep/wake thresholds for OB gamma and EMG power that were independently determined during a previous sleep/wake session in the home cage. (B) In grey, distribution of gamma (left) and EMG (right) power during sleep and wake in the home cage. In black, the distribution during the test session, including the freezing periods. Gamma power during the test session is systematically classified as wake, whereas the EMG power during freezing periods drops below the sleep/wake threshold. (C) Averaged OB gamma power (left) and EMG power (right) triggered on two types of transitions from mobility to immobility: the wake-to-sleep transition and the active-to-freezing transition. OB gamma power drops when the animal falls asleep but not when the animal freezes. EMG power shows similar changes during freezing and sleep onset (*n* = 6, error bars: s.e.m.). EMG, electromyography; OB, olfactory bulb.

The example session shown in Fig 6A illustrates the strong expected drop in EMG power during freezing periods, sometimes below the sleep/wake threshold independently determined during a previous home cage session. Although the EMG power is indeed on average higher during freezing than during the sleep state, certain freezing time points can be misclassified as sleep (Fig 6B). Moreover, EMG shows a similar drop in power at freezing and sleep onset (Fig 6C). In sharp contrast, OB gamma power remains systematically above the sleep/wake threshold (Fig 6B). Gamma power triggered on freezing onset shows that the variable is independent of this abrupt change in movement (Fig 6C).

Freezing is a behaviour that dissociates complete immobility from sleep, allowing us to clearly show that OB gamma power is tracking transitions from wake to sleep and not from mobility to immobility. EMG, in contrast, is an unreliable marker for sleep scoring when animals are susceptible to display immobility during wakefulness.

### The different dynamics of waking up and falling asleep

Our automated sleep scoring method classifies all data points into periods of wake, NREM, and REM. Thus considered, changes in vigilance are reduced to a sequence of steady states, yet a crucial aspect of sleep concerns the transitions between states. Here we show that these transitions can be studied by visualizing moment-to-moment variations in brain state as a moving point in the previously described phase space (S1 Movie). The trajectories can then be studied using tools from statistical physics.

**Fig 7:**
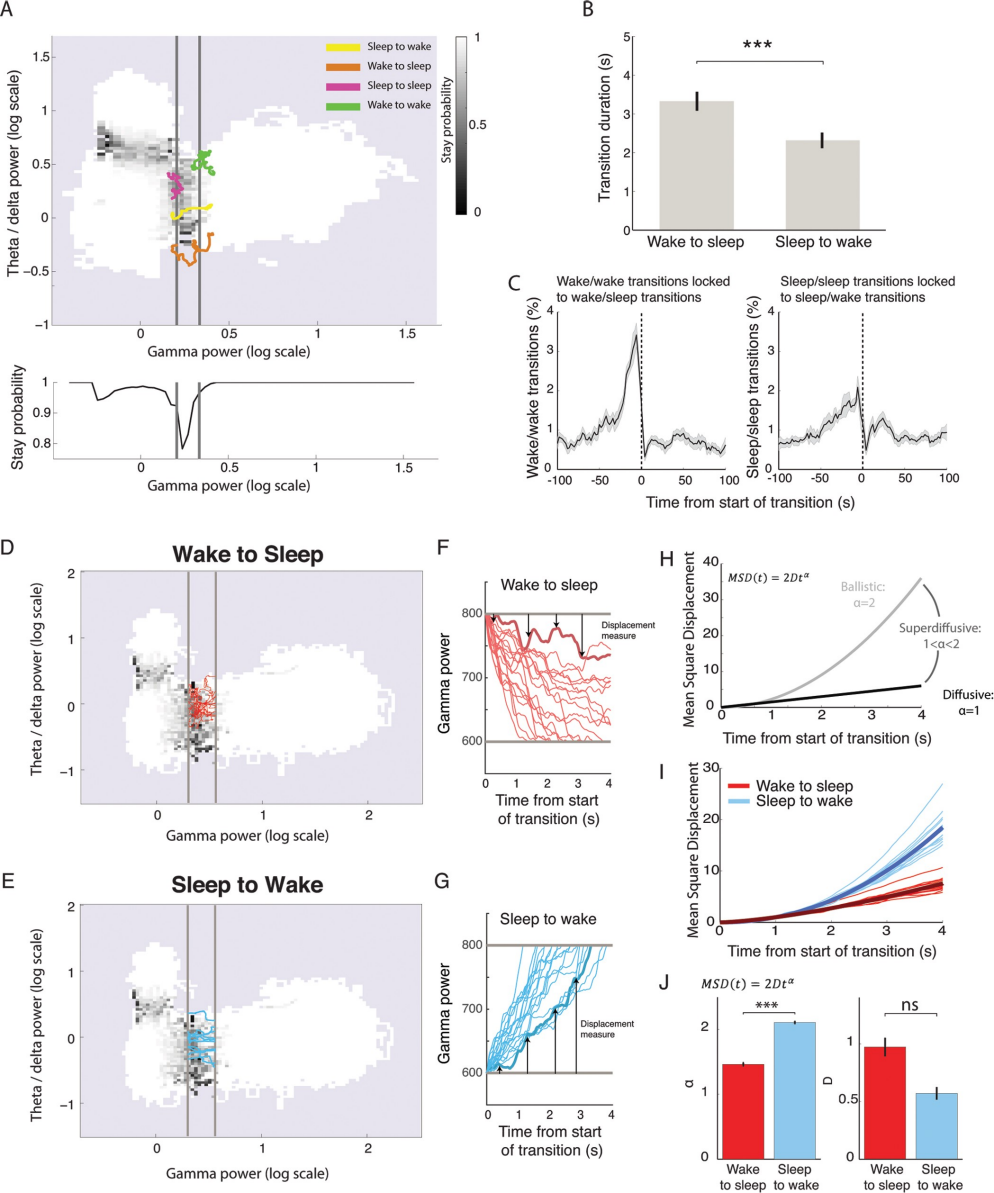
Analysis of phase space trajectories to study the dynamics of state transitions. (A) Phase space map of the stay probability, i.e., the probability of remaining in the current state after 3 s. Far from state boundaries, stay probability equals 1 (white), indicating that this part of the phase space is highly stable; the frontiers between states are characterised by low stay probability (black). Averaging the stay probability along the gamma power axis (bottom panel) reveals a strong dip in stay probability that is identified as the transition zone between sleep and wake (grey lines). Trajectories around the transition border illustrate full transitions (sleep to wake and wake to sleep) and aborted transitions (sleep to sleep and wake to wake). (B) Traversal time of the transition zone showing that wake-to-sleep transitions are significantly longer than sleep-to-wake transitions (paired *t* test: *p* = 0.0007, *n* = 15; error bars: s.e.m.; *** *p* < 0.001). (C) Cross-correlation of aborted transitions’ times relative to true transitions. Wake-to-sleep transitions are more tightly coupled to aborted transitions from wake than sleep-to-wake transitions to aborted transitions from sleep (error bars: s.e.m.). (D, E) Phase space of stay probability with transition zones shown in grey with 10 randomly chose transition trajectories from wake to sleep (D) or from sleep to wake (E). (F, G) Gamma power as a function of time for wake-to-sleep (F) and sleep-to-wake (G) trajectories. Arrows show how displacement is measured for the trajectory shown in thicker line: at each time point, the distance to the transition zone is calculated. The square of this value yields the MSD. (H) Theoretical relationship between MSD and time for three regimes of motion: ballistic, superdiffusive, and diffusive. (I) MSD for sleep-to-wake and wake-to-sleep trajectories as a function of time after normalisation by D for clarity. Thick line: average over all mice (*n* = 15). Note the clear separation of the two sets of lines. (J) Values of alpha and D obtained after fitting the MSD curves (F). Note that alpha is significantly increased for sleep-to-wake transitions relative to wake-to-sleep transitions. (paired *t* test: alpha: *p* = 7E-11, D: *p* = 0.2, *n* = 15, *** *p* < 0.001). MSD, mean square displacement; ns, not significant.

Shifting to this finer timescale, we replaced the previously used threshold with a transition zone, consistent with many experts arguing that there is no single time point corresponding to the onset or offset of sleep [26]. For each point in phase space, the probability of remaining in the current state (the ‘stay probability’) is calculated, revealing highly stable zones far from the thresholds (Fig 7A, white) and zones of instability in which the state of the brain is changing close to the thresholds (Fig 7A, black). Two transition zones naturally emerged along the gamma power axis between sleep and wake states and the theta/delta ratio axis between REM and NREM. In the rest of the study, we focus on the sleep/wake transition. The sleep/wake transition zone can be identified by measuring the average stay probability along the gamma power axis (Fig 7A, lower panel), which displays a strong dip between sleep and wake states. We identify this low stay probability region as the transition zone, which is shown between the grey lines in Fig 7A. We propose three measures based on this transition zone to study the dynamics of state change and illustrate that it allows for novel characterisation of the sleep/wake transition.

First, we defined ‘true transitions’ as a full crossing of the transition zone. We found that the duration of this crossing depended on the direction. Wake-to-sleep transitions were significantly slower than sleep-to-wake transitions (Fig 7B).

Second, we also defined ‘aborted transitions’ during which the brain state enters this transition zone but does not make a full crossing. Aborted wake/wake transitions were more frequent than sleep/sleep transitions (*p* = 0.005, *n* = 15, paired *t* test). These aborted wake/wake transitions tended to be strongly grouped around the 30 s preceding complete wake/sleep transitions, whereas sleep/sleep transitions were only weakly related in time to sleep/wake transitions (Fig 7C). This indicates that falling asleep is preceded by many aborted transitions contrary to waking up.

Finally, we adopted a dynamic approach to analyse these trajectories. Differences in transition durations (Fig 7B) from wake to sleep and sleep to wake can be interpreted as differences in speed only if the movement is well described by ‘ballistic’ motion. Ballistic motion indicates that time, speed, and distance are linearly related, as when an apple falls to the ground (Fig 7H, grey). This is not true for all types of motion, particularly those describing stochastic processes such as diffusive motion, as when a grain of pollen diffuses on a bowl of water (Fig 7H, black). To evaluate whether the observed motion is closer to the ballistic or diffusive regime, the mean square displacement as a function of time can be modelled thus:

$$MSD(t)=\langle {\left(x(t+\tau )-x(\tau )\right)}^{2}\rangle =2{Dt}^{\alpha }$$

Theory of Brownian motion indicates that when alpha = 2, the movement is ballistic, and for alpha = 1, the movement is diffusive. For alpha < 2 and > 1, the movement is described as superdiffusive. Transitions with essentially similar dynamics but different speeds are described by the same value of alpha but different values of D. On the other hand, a change in the value of alpha argues for different transition dynamics and thus potentially different underlying mechanisms.

Fig 7D–7G compares the same number of transition trajectories from wake to sleep (top) and from sleep to wake (bottom). The wake-to-sleep trajectories appear much more convoluted and tile the plane more densely than the sleep-to-wake trajectories, suggesting that their movement is more diffusive. The mean square displacement curves for both transitions types clearly segregate into two groups with a faster rise time for the sleep-to-wake transition, as expected (Fig 7I). We can now ask what the underlying difference between these two curves is by fitting them to the simple equation as defined above (mean R^2^ = 0.996, all R^2^ larger than 0.988). Fitting reveals that the motion from sleep to wake is ballistic (mean alpha = 2.1), whereas the motion from wake to sleep is more diffusive (mean alpha = 1.47) (Fig 7J).

Overall, using the phase space approach, we can clearly demonstrate a strong and novel dichotomy between sleep–wake and wake–sleep transitions. In particular, the awakening transition is fast and ballistic, whereas the process of falling asleep is slower and more stochastic, preceded by multiple failed attempts to transition. Finally, the frequent aborted sleep/sleep transitions that do not precede actual awakenings may be linked to subthreshold arousal phenomena such as the cyclic alternating pattern [27].

### REM and NREM sleep signatures in OB activity

When considering previous observations, the most likely explanation is that OB activity can act as an index of vigilance state because it reflects the ongoing neuromodulatory state of the brain, probably because of the numerous projections coming from regions in the brain stem known to regulate sleep and wake (see Discussion). If this reasoning is correct, OB activity should also be different between REM and NREM sleep, given that they show very different neuromodulatory profiles.

**Fig 8:**
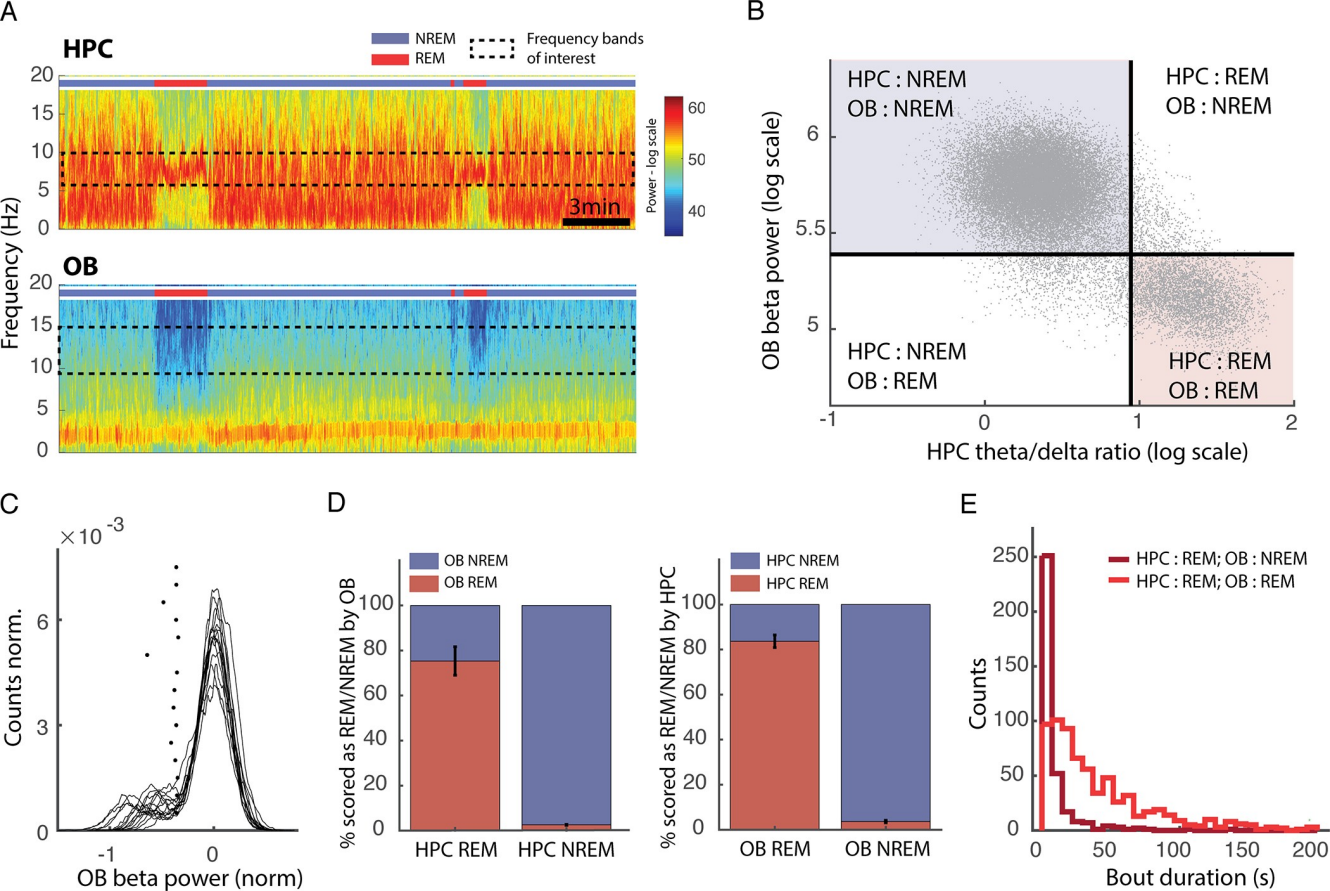
Reduction of OB beta activity during REM sleep allows good NREM/REM discrimination. (A) Spectrograms of OB (top) and HPC (bottom) activity. The coloured bar indicates the state of the animal defined using OB and HPC activity. Note that during REM periods, defined by strong theta activity in the HPC, there is a drop in a wide band from 10 to 20 Hz in the OB (bar: 3 min). (B) Correlation of OB beta power and HPC theta/delta ratio power for the same mouse as in A. Black: automatically determined thresholds. Dots in the upper left and lower right-hand corner are identically classified by both approaches. (C) Distributions of beta power in the OB during sleep with automatically determined thresholds (*) (*n* = 15). (D) Percentage of overlap of REM and NREM scored using the HPC (left) of the OB (right) with the other methodology: OB and HPC, respectively (*n* = 15, error bars: s.e.m.). (E) Distribution of bout duration for REM periods identified by both HPC and OB scoring (light red) or identified only by HPC scoring (dark red). Note that bouts missed by the OB scoring are systematically very short. HPC, hippocampus; NREM, non-REM; OB, olfactory bulb; REM, rapid eye movement.

Accordingly, we observed that beta range activity (10–20 Hz) in the OB was much weaker during REM sleep than NREM sleep (Fig 8A). Activity in this band during sleep clearly segregated into two levels that strongly correlated with HPC theta/delta ratio (Fig 8B). The distribution of OB beta power is similar to that described for the HPC theta/delta ratio: a clear peak of values corresponding to NREM sleep and a broader slab of values for REM sleep. We therefore placed an automatic threshold to separate the two states using the same methodology as for the HPC and applied the rule that beta power below threshold corresponded to REM sleep and above threshold to NREM sleep. We then evaluated the agreement with data scored using HPC LFP. We found that there was a good agreement between the two methods: 97% of NREM periods and 75% of REM periods, as defined by the HPC, were correctly identified using OB scoring (Fig 8D). REM sleep defined by OB scoring corresponded to HPC-defined REM sleep in 83% of cases.

We next more closely investigated the periods of REM sleep (25%) identified by the HPC that were misidentified as NREM based on the OB beta power (Fig 8D). We found that in fact some REM bouts were missed entirely when using the OB beta method, accounting for 14% out of the 25% error rate, and they tended to be particularly short (Fig 8E). The finding that OB beta activity can be used to identify REM sleep opens the possibility of sleep scoring using a single wire in the OB to track wake, REM, and NREM sleep, with most errors being restricted to short bouts. This modulation of another OB rhythm by brain state confirms our hypothesis that activity in the brain region is highly sensitive to neuromodulatory changes.

### OB activity tracks depth of anaesthesia and predicts reaction to future stimulation

Given the remarkable link between OB oscillations and sleep/wake states, we asked whether OB gamma could provide an even more general indicator of vigilance by investigating the impact of anaesthesia.

**Fig 9:**
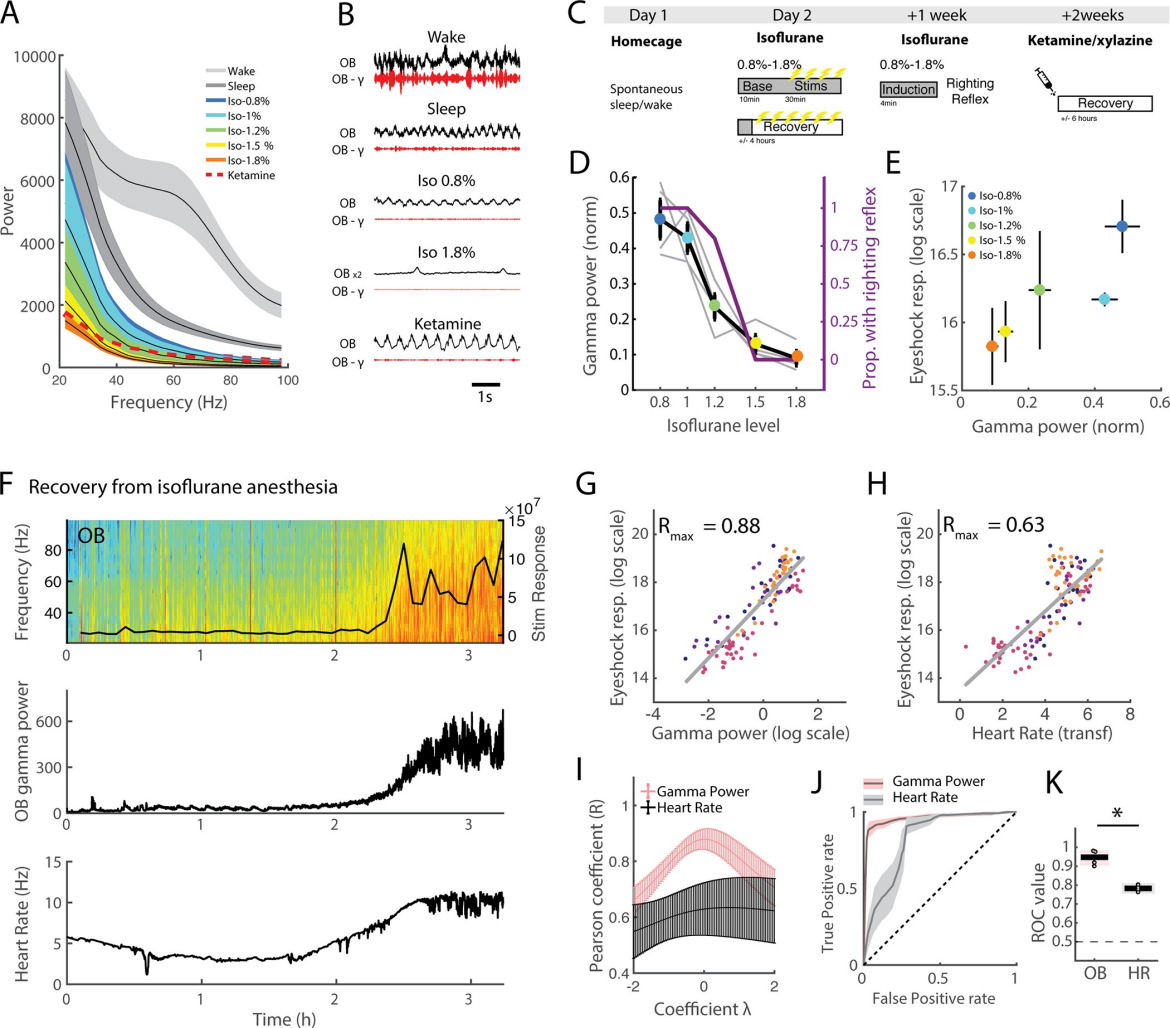
Link between OB activity in the gamma band and depth of anaesthesia. (A) Spectra from wake/sleep and anaesthetised states (*n* = 4 for isoflurane [‘iso’], *n* = 3 for ketamine/xylazine ['ketamine'], error bars: s.e.m.). (B) Example data across states showing raw and filtered (50–70 Hz) OB signals. Raw data from 1.8% isoflurane is scaled up by a factor 2 for visibility. The same scale is always used for gamma power. (C) Schematic of protocol. Mice were first recorded in their home cage to establish the sleep/wake threshold. Then, under 5 levels of isoflurane anaesthesia, a baseline of 10 min activity was recorded, after which mice received 2 V eye shock stimulations to evaluate reaction levels to noxious stimulation. At the end of the protocol (recovery), mice were recorded until they regained full mobility. One wk later, mice were re-anaesthetised at the same 5 isoflurane concentrations, and after the induction period, their righting reflex was scored. Finally, 1 wk later, mice were injected with a ketamine/xylazine cocktail and recorded until they regained full mobility. (D) Mean OB gamma power normalised to sleep levels (1 = mean NREM gamma level) during baseline period and proportion of mice with righting reflex as a function of isoflurane concentration (*n* = 4, error bars: s.e.m.). (E) Mean stimulus response to 2 V eye shock evaluated with head acceleration as a function of OB gamma power normalised to sleep levels (1 = mean NREM gamma level) during baseline period. (F) Example of recovery session for one mouse. At time 0, isoflurane delivery was stopped. The top panel shows OB spectrogram and the response to eye shock stimulation, the middle panel shows OB gamma power, and the bottom panel shows the heart rate. Note the rise in gamma power and heart rate as the animal begins to respond to stimulation. (G) Stimulus reaction throughout recovery session for all mice (each mouse is a different colour) as a function of log-scaled OB gamma power normalised to sleep (0 = mean NREM gamma level). OB gamma is plotted with a log scale, as this gave the optimal correlation as evaluated in panel I. (H) Stimulus reaction throughout recovery session for all mice (each mouse is a different colour) as a function of heart rate. Heart rate has been transformed to obtain the optimal correlation as evaluated in I. (I) Pearson correlation coefficient for OB gamma and heart rate correlated with stimulus reaction as a function of lambda, the transformation parameter of the Box-Cox transformation. This continuous transformation allows us to find the best monotonic transformation of data to improve the validity of the Pearson correlation coefficient, which assumes linearity between the two variables, given that this relationship may be in fact nonlinear (see Materials and methods for details). Note that OB gamma is systematically better correlated than heart rate. The peak correlation is used to choose the lambda coefficient, which is applied to transform data in G and H (*n* = 4, error bars: s.e.m.). (J) ROC curve for OB gamma and heart rate prediction of whether stimulus reaction is classified as ‘sedated’ or ‘aroused’ according to threshold. The black dotted line indicates the curve expected by chance. (K) Area under the ROC curve for OB gamma and heart rate (‘HR’). The black dotted line shows the expected chance level. The black line at 0.5 shows the expected chance level (Friedman test χ^2^(3) = 4, *p* = 0.045; *** *p* < 0.05). NREM, non-REM; OB, olfactory bulb; ROC, receiver operating characteristic.

We evaluated the impact of isoflurane and ketamine/xylazine anaesthesia on OB oscillations and linked these changes to two classical measures of anaesthetic depth: response to noxious stimulation and loss of righting reflex (Fig 9C). The 50–70 Hz band that is prominent during wake is suppressed during both sleep and anaesthesia (Fig 9A and 9B). Under anaesthesia, we in fact observed a steady decrease in global power with depth of anaesthesia, which can be fully tracked even after restricting analysis to the 50–70 Hz band. This suggests that measuring gamma power not only provides information about the transition from wake to sleep to anaesthesia but could also be used to monitor depth of anaesthesia.

We first imposed different levels of steady-state isoflurane anaesthesia and measured the corresponding activity in the OB. We found that OB gamma power tracks isoflurane concentration increase concomitantly with the loss of righting reflex (Fig 9D). During anaesthesia, we electrically stimulated the eyelids of the mice to provide a reproducible and time-locked measure of their responsivity to noxious stimulation and evaluated their motor responses every 2 min. Predictably, response to stimulation was reduced as isoflurane concentration increased, and the average gamma power tracked this change closely (Fig 9E). These results show that the steady-state value of OB gamma under anaesthesia is a good correlate of two behavioural measures of anaesthetic depth: the righting reflex and response to stimulation.

We next asked whether OB gamma power can follow dynamic changes and monitor in real time the depth of anaesthesia. We addressed this question by evaluating whether OB gamma power could predict stimulation responsivity during anaesthesia recovery. Once mice had reached the deepest level of isoflurane anaesthesia, we stopped isoflurane delivery and left them to regain consciousness in their home cage whilst continuing to stimulate at regular intervals so as to track their arousal level (Fig 9F). As can be seen in Fig 9F, as mice began to respond to stimulation and regained motility, the OB gamma power increased with a strikingly similar time course.

We found that OB gamma activity during the 3 s prior to eye shock stimulation (excluding 500 ms prior to stimulation to avoid spectral leakage) can predict the animal’s future response. First of all, the gamma power in the 3 s before the shock correlated very strongly with the animal’s response (Fig 9G). To evaluate the relevance of this observation, we compared it with one of the main physiological markers of anaesthetic depth, heart rate, and found that the OB gamma was a more reliable correlate of stimulation response. Since the relationships between these variables and stimulation responsivity may follow an unknown nonlinear function, we used the Box-Cox transformation, which explores a smooth range of monotonic functions to find the transformation that yields a maximally linear relationship. This allows a fair comparison of correlation strength between our two variables despite potential nonlinearities in the data. Taking the maximal correlation after transformation, heart rate also correlated well with stimulus reactivity (Fig 9H); however, OB gamma power systematically showed a stronger correlation (Fig 9I).

As can be seen in Fig 9F, reactivity to stimulus is clearly bimodal during recovery, allowing us to place a natural threshold between ‘sedated’ and ‘aroused’ responses. By using receiver operating characteristic (ROC) curves, we showed that OB gamma power before stimulation almost perfectly predicts the state of anaesthesia based on the binarised response to noxious stimuli (Fig 9J and 9K). As for the correlation measures, OB gamma was a better predictor than heart rate (Fig 9J and 9K).

These results demonstrate that OB activity is as sensitive to levels of vigilance and reactivity during anaesthesia-induced sedation as it is to natural changes during sleep and wake. The OB gamma power can therefore also be used to track depth of anaesthesia with high accuracy and in real time, since it can predict and in fact anticipate the future reaction to stimulation.

## Discussion

Since its invention, EEG has been the tool of choice to attempt to monitor vigilance states using brain activity. This endeavour has been most successful in identifying wake, sleep, and the substages of sleep, and studying their microstructure has become an essential aspect of understanding the brain in health and disease [27]. Therefore, sleep scoring is the essential first step in any study of global brain states. Here we establish for the first time, to our knowledge, a methodology for addressing this issue with brain signals alone. We found that gamma oscillations in the OB allow us to continuously discriminate sleep from wake, substituting muscular activity or body movements, which are required in all the other sleep scoring methods. They can also be used to transpose the evolution of brain states into a phase space in which the fine dynamics of state transitions can be analysed. This methodology also captures the modification of vigilance state induced by anaesthesia. We demonstrated that OB gamma activity can predict and in fact anticipate the response to noxious stimulation and can therefore be used as a real-time tracker of anaesthetic depth. Altogether, this shows that OB activity can be used to track vigilance states in real time in a wide range of situations with high accuracy without having to rely on muscular or other body-related measures.

There is a long history of the study of the link between activity in the olfactory system and brain states, which has in particular focused on the correlation of OB oscillations and arousal level. The earliest recordings made in the OB by Adrian identified the fast oscillations under anaesthesia [28] but already noted an ‘awakening reaction’ as anaesthesia gradually lightened [29]. He therefore suggested that the OB oscillations are damped by anaesthetic drugs and increased by waking activity. Later, ‘arousal discharges’ (34–48 Hz) were identified in the cat OB that were potentiated by attention and disappeared during sleep and anaesthesia [30,31]. These global changes in OB oscillatory profile depending on level of arousal have been described and modelled as a hierarchy of attractors with increasing potential for fast oscillatory activity ranging from anaesthesia to seizure [32]. More recent studies have confirmed these changes in OB activity related to sleep states [17] and anaesthesia [33,34].

The novel sleep scoring method we propose relies on activity recorded in the HPC and the OB only. Implantation of electrodes for recording LFP in these two areas is easy to achieve because they both show robust oscillations in the theta and gamma ranges respectively. After implantation, the method is fully automated and therefore removes the time-consuming steps of manually scoring the data set in its entirety or in part to calibrate semiautomatic algorithms. We have shown that this method for sleep scoring is robust to slight changes in implantation site, across days, and between different animals. This robustness to implantation site may seem surprising given the high spatial heterogeneity of gamma oscillations in the HPC [35] and cortex [36]. However, in the OB, oscillations in the gamma range are highly coherent across large areas, despite fast-varying phase shifts [37]. Finally, the method is applicable to multiple strains of mice and after drug injection. It therefore allows easy comparison between mice and throughout time and should enhance comparability of data sets from different laboratories.

Beyond the technical ease of use, this method also provides a promising framework for the study of global brain states. Using activity recorded in the brain and not muscle activity allows us to track sleep/wake activity independently of movement, as we demonstrated with the example of freezing, a period of tonic immobility linked with fear expression. This could provide a heretofore-lacking methodology to study phenomena such as REM without atonia induced in lesion studies [38].

Finally, gamma activity in the OB is a variable with fast dynamics that allows us to study fine timescale transitions not accessible to other, slower sleep-related oscillations such as delta power. The mechanisms that control the transitions between sleep and wake have been extensively studied [39], but how they relate to changes in more global physiological variables is an open and essential question. The process of falling asleep has been described using behavioural, physiological, and electrophysiological variables as a progressive sleep onset period in humans [26]. After awakening, the prolonged deficits in performing certain tasks, named sleep inertia [40], has been described in humans and in rats, and mice OFF periods were observed up until 5 min after awakening [41]. All these studies demonstrate that transitions should not be considered as points in time but instead as extended periods justifying our use of transition zones.

We advocate constructing phase spaces with electrophysiological variables (OB gamma and HPC theta/delta ratio) capable of tracking high-speed changes in the brain state. This phase space remains stable in time and is reproducible between animals, which is a crucial step towards characterising the dynamical evolution of sleep states. Tracking activity within this phase space allows us to model changes in brain state with the rich repertoire of tools taken from statistical physics. The efficiency of this approach is illustrated by its ability to capture the deep asymmetry between falling asleep and waking up [42]. Indeed, falling asleep can be modelled as a slower, diffusive process, whereas waking up corresponds to a faster, ballistic change. This is consistent with the fine evolution of neuronal activity at transition times in brain stem and hypothalamic nuclei responsible for the sleep/wake switch that show faster dynamics upon awakening than falling asleep [43–46].

We also showed that OB activity in the beta range (10–20 Hz) can be used to identify REM and NREM sleep with high reliability. The rare errors of discrimination concern particularly short REM bouts. Given the strength of HPC theta in the rodent allowing for clear identification of REM sleep, we still recommend recording in the HPC whenever possible. However, the use of a single wire in the OB to discriminate sleep and wake (OB gamma) and REM and NREM sleep (OB beta) could be a useful strategy when using bulky headstages, such as head-mounted microscopes, that may block access to the HPC.

Although our main focus is on the ability of OB gamma to discriminate sleep from wake, since this was an important issue in the field of sleep scoring, we also show that it can be used to track anaesthetic depth in real time. Steady-state OB gamma power systematically decreased as we induced deeper levels of anaesthesia by increasing isoflurane concentration and therefore was strongly correlated with two different measures of anaesthetic depth: the loss of righting reflex and the response to eye shock stimulation. Moreover, OB gamma power also closely tracked the changing level of arousal as mice recovered from anaesthesia. It could predict the reaction level to a future stimulation with remarkable accuracy and systematically outperformed another common marker of anaesthesia level, heart rate. These results are particularly striking, since, to date, we lack a clear marker to monitor depth of anaesthesia in surgical settings [47,48], and in particular, the most commonly used indicator, the bispectral index (BIS), fails when ketamine is administered [49]. The similarity of OB activity under isoflurane and ketamine/xylazine anaesthesia therefore makes it a remarkably polyvalent marker to track depth of anaesthesia.

Altogether, these observations raise the question as to why, compared with other areas, the OB is so well suited to tracking global changes in brain state. We propose that the OB is ideally situated to produce oscillatory activity that is exquisitely sensitive to brain state, since it receives massive inputs from most of the neuromodulator systems and notably those involved in the control of vigilance: cholinergic [50,51], hypocretinergic/orexinergic [52], and noradrenergic [53]. Moreover, receptors of these neuromodulatory systems are strongly expressed in the OB [54,55]. In turn, the different neuromodulators have been shown to modulate gamma oscillations [34,56–58]. More specifically, it is widely agreed upon that the mechanism for gamma generation in the OB relies on the dendrodendritic interactions between mitral/tufted cells and the local granule cells that provide recurrent and lateral inhibition [59–61]. The strength of this inhibition has been shown to be modulated by global brain state via a cholinergic mechanism [62]. This pattern of a macroscopic physiological parameter serving to amplify fluctuations in neuromodulatory tone is reminiscent of a recent method that has been proposed to continuously track vigilance states by recording pupil diameter [63]. It was shown that pupil diameter follows the activity of cholinergic and adrenergic activity in the cortex [64]. Finally, the difference of OB beta power between REM and NREM strongly argues in favour of OB activity providing a general readout of the global neuromodulatory state of the brain.

It is possible that OB activity is not just an index of arousal but may actually take an active part in its regulation. In seminal work, Arduini and Moruzzi studied acute *cerveau isolé* cats and found that stimulation of the olfactory system by blowing air into the nostrils led to arousal, contrary to visual stimulation [65]. These results did not depend on the use of odour stimulations, which suggests that the role of the olfactory system in arousal goes beyond that of the olfactory sensory modality per se. Moreover, perturbation of the olfactory system leads to changes in sleep structure [66]. These observations have led to the proposal that the OB may actively participate in a secondary arousal system [67,68] linked to limbic areas that complements the ascending reticular activating system [69].

Even without a direct influence on the regulation of vigilance states, OB gamma activity could also have a functional role in regulating reactions to sensory stimuli during sleep. The functional importance of OB gamma oscillations was first brought to light by the seminal work of W. J. Freeman, in which analysis of the spatial spread of odour-induced gamma amplitude revealed that these patterns changed with learning and were related to the behavioural meaning of the odour [70,71]. Since, gamma oscillations in the olfactory system have also been shown to be modified during learning [72], and their perturbation by pharmacological [73] or genetic [74] tools interfere with odour processing. The importance of gamma oscillations in sensory processing leads us to hypothesise that their suppression during sleep could provide a mechanism for sensory gating, as has been suggested previously [75]. During sleep, cortical responses to sensory stimulation are still observed [76–78], but they are not accompanied by conscious experience of the stimuli or behavioural reactions. Olfaction is the only sensory system in the mammalian brain that does not travel through the thalamic relay before reaching the cortex. The thalamus is thought to play an important role in information gating during sleep [79]. Therefore, alternative mechanisms must be at play within the olfactory system. A global and continuous shift of the dynamical state of the olfactory system with arousal has been suggested [32], as well as gating within the piriform cortex [80]. We suggest that direct suppression of local gamma activity in the OB necessary for information processing may also play a role.

The question of whether activity in the OB has a functional role in modifying sensory processing during sleep or on sleep regulation itself would require further study. Nevertheless, the results presented here show that recording OB oscillations is an attractive strategy for monitoring a wide range of vigilance states in natural situations independently of motor confounds. Our methodology provides the possibility of tracking levels of vigilance from wakefulness to anaesthesia in real time and of sleep scoring independently of movement and could also provide a potential alternative to HPC theta for discriminating REM from NREM sleep. In conclusion, OB gamma activity clearly opens an unprecedented window onto levels of vigilance.

## Materials and methods

### Ethics statement

All behavioural experiments were performed in accordance with the official European guidelines for the care and use of laboratory animals (86/609/EEC) and in accordance with the Policies of the French Committee of Ethics (Decrees n° 87–848 and n° 2001–464). Animal housing facility of the laboratory where experiments were made is fully accredited by the French Direction of Veterinary Services (B-75-05–24, 18 May 2010). Animal surgeries and experimentations were authorised by the French Direction of Veterinary Services for KB (14–43).

### Subjects and surgery

A total of 15 C57Bl6 male mice (*Mus musculus*), 4 Gad2-IRES-Cre knock-in C57BL/6J Rj, 3 DBA/2Rj, and 2 C3H/HeNRj mice were used in this study. Mice were housed in an animal facility (08:00–20:00 light), 1 per cage after surgery.

At 3–6 mo of age, mice were implanted with electrodes (tungsten wires) in the right OB (AP +4, ML +0.5, DV −1.5) and in the right CA1 hippocampal layer (AP −2.2, ML +2.0, DV −1.0). Six of these mice were also implanted with a hooked EMG wire in the right nuchal muscle. Six mice were also implanted in the right prefrontal cortex (AP +2.1, ML +0.5, DV −0.5) and parietal cortex (AP −1.7, ML +1.0, DV −0.8). One mouse was recorded with a 16-site linear probe (100 μm spacing, Neuronexus Tech, Ann Arbor, MI, United States). Four mice were also equipped with ECG wires to monitor heart rate. Two highly flexible coiled wires were sutured above and below the heart and then travelled under the skin to be fixed to the headstage and preamplified with the other recording wires. During recovery from surgery and during all experiments, mice received food and water ad libitum. Recordings began 1 wk after surgery.

Signals from all electrodes were recorded using an Intan Technologies amplifier chip (RHD2216, sampling rate 20 KHz). LFPs were sampled and stored at 1,250 Hz. Analyses were performed with custom-made Matlab programs, based on generic code that can be downloaded at http://www.battaglia.nl/computing/ and http://fmatoolbox.sourceforge.net/.

### Fear conditioning

The protocol has been previously described [81]. Habituation and fear conditioning took place in context A consisting of a square transparent plexiglass box in a black environment with a shock grid floor and cleaned with ethanol (70%) before and after each session. Test sessions were performed in context B consisting of cylindrical transparent plexiglass walls with a grey plastic floor placed in a white environment and cleaned with acetic acid (1%) before and after each session.

To score freezing behaviour, animals were tracked using a homemade automatic tracking system that calculated the instantaneous position of the animal and the quantity of movement defined as the pixelwise difference between two consecutive frames. The animals were considered to be freezing if the quantity of movement was below a manually set threshold for at least 2 s.

On day 1, mice were submitted to a habituation session in context A, in which they received four presentations of the CS− and of the CS+ (total CS duration, 30 s; consisting of 50 ms pips at 0.9 Hz repeated 27 times, 2 ms rise and fall; pip frequency, 7.5 kHz or white noise, 80 dB sound pressure level). Discriminative fear conditioning was performed on the same day by pairing the CS+ with a US (1 s foot shock, 0.6 mA, 8 CS+ US pairings; intertrial intervals, 20–180 s). The onset of the US coincided with the offset of the CS+. The CS− was presented after each CS+ US association but was never reinforced (8 CS− presentations; intertrial intervals, 20–180 s). On day 2, conditioned mice were submitted to a test session in context B during which they received 4 and 12 presentations of the CS− and CS+, respectively.

### Anaesthesia experiments

For isoflurane experiments, anaesthesia was initiated by exposing mice to a mixture of 3% isoflurane and oxygen in an induction chamber (MSS international, IsoTec3). The mouse was then maintained under constant isoflurane in oxygen at the desired concentration with only the nose placed in a small mask. Whenever isoflurane level was changed, recordings or experiments commenced at least 4 min later to allow stabilisation. Throughout the experiment, mice were in contact with a warm pad.

For ketamine experiments, mice were injected with a xylazine (10 mg/kg) ketamine (100 mg/kg) mixture at the beginning of the experiment and then recorded continuously whilst in contact with a warm pad.

Noxious stimuli were delivered using bilateral eyelid shock with implanted silver wires. This allowed us to evaluate the response levels to a precisely timed and reproducible stimulation, contrary to manual tail pinch, for example. We used 2 V stimulation in all analyses.

Righting reflex was measured by removing the mouse quickly from the induction box after 4 min of isoflurane delivery and placing it on its back on a flat surface. If the mouse righted itself (4 paws on the tabletop) within 10 s, it was scored as having retained its righting reflex.

### Histological analysis

After completion of the experiments, mice were deeply anaesthetised with ketamine/xylazine solution (10%/1%). With the electrodes left in situ, the animals were perfused transcardially with saline (approximately 50 ml), followed by approximately 50 ml of PFA (4 g/100 mL). Brains were extracted and placed in PFA for postfixation for 24 h, transferred to PBS for at least 48 h, and then cut into 50-μm-thick sections using a freezing microtome and mounted and stained with hard set vectashield mounting medium with DAPI (Vectorlabs).

### Bimodality quantification

Bimodality was quantified by fitting a mixture of two normal distributions and evaluating either Ashman’s D [82], $D=\sqrt{2}\frac{\left|\mu_{1}-\mu_{2}\right|}{\left|\sigma_{1}+\sigma_{2}\right|}$ where D > 2 is required for a clean separation or the overlap of the two distributions.

### Automatic sleep scoring algorithm

LFP recordings from the OB were filtered in the gamma (50–70 Hz) band and instantaneous amplitude derived from the Hilbert Transform. This time series was then smoothed using a 3 s sliding window (Fig 1D), and the distribution of values could be fit with a mixture of two Gaussian distributions. To maximise the probability of correct classification, the threshold between sleep and wake should be defined as the intersection of these two distributions. This value, however, depends on the amplitude of the two distributions and therefore on the ratio of sleep and wake recorded. To establish a threshold independent of this ratio, the two distributions are normalised to each have area one (Fig 2Bi, right), and the intersection of these distributions is used. Values inferior to this value are classified as sleep and those superior as wake. Periods of sleep and wake shorter than 3 s were merged into the surrounding periods to avoid artificially short epochs. Then, LFP recordings from the HPC restricted to the sleep periods defined above were filtered in the theta (5–10 Hz) and delta (2–5 Hz) bands and instantaneous amplitude derived from the Hilbert transform. The ratio of the theta and delta powers was smoothed using a 2 s sliding window, and the distribution of values was fit by a single normal distribution that accounted for the NREM data points (low theta/delta ratio). The REM/NREM threshold was placed at the value of theta/delta ratio, above which the residuals systematically explained more than 50% of the actual data (Fig 2Bii). Periods of NREM and REM shorter than 3 s were merged into the surrounding periods to avoid artificially short epochs.

### Automatic EMG scoring

Automatic EMG scoring was performed in a similar fashion to automatic OB gamma power scoring. EMG data was filtered in the 50–300 Hz band and instantaneous amplitude derived from the Hilbert transform. This time series was then smoothed using a 2 s sliding window, and the distribution of values could be fit with a mixture of two normal distributions. The intersection of these two distributions, once normalised, provided the sleep–wake threshold. The procedure for identifying REM/NREM sleep is identical to that used in the OB-based sleep scoring algorithm as described above.

### Manual sleep scoring

Automatic scoring was performed independently by two experimenters using a homemade Matlab GUI. The scorers were provided with EMG (raw, filtered in the 50–300 Hz band and smoothed instantaneous amplitude) and HPC (raw, low-frequency spectrogram and smoothed instantaneous theta-to-delta ratio). Scorers were presented with 3 s windows of data that they had to identify either as NREM, REM, or Wake. When two states were identified in the same window, it was scored as the state occupying more than 50% of the epoch.

### Evaluation of overlap of scoring methods

The percentage agreement between methods is calculated for each state and shown in the relevant figures. Given that average agreement can be potentially misleading, we also used the confusion matrix to calculate Cohen’s κ [83], defined as the following:

$$K=\frac{P_{o}-P_{e}}{1-P_{e}}$$

with

$P_{o}=\sum_{i=1}^{3}p_{ii}$, where *p*_*ii*_ is the probability that both methods classify data as the identical state i (REM, NREM, wake).

$P_{e}=\sum_{i=1}^{3}p_{1i}\times p_{2i}$ where *p*_1*i*_ and *p*_2*i*_ are the independent probabilities that methods 1 and 2 will classify data as state i.

We applied the same criteria as used in [10] to evaluate the quality of the agreement (Table 2).

**Table 2:** 

Quality of agreement	Cohen’s κ
Almost perfect	>0.81
Substantial	0.8–0.61
Moderate	0.6–0.41
Fair	0.4–0.21
Slight	0.2–0
Poor	<0

### Phase space analysis

Step 1: generate stay probability map

- For each time step in the data set, calculate the time to the next transition.
- Binarise this data into ‘stay’ (1) and ‘leave’ (0). If the duration is above the fixed ‘stay period’ (3 s, in our case), then this time point is considered as remaining within its state and is given the value 1; otherwise, it is given the value 0.
- Bin each time step according to its position in the phase space, i.e., its instantaneous OB gamma power and HPC theta/delta ratio.
- For each coordinate of the phase space, calculate the average of the binarised stay/leave values; this yields the stay probability map.

Step 2: identify transition zones

- Along the axis of interest (OB gamma power for sleep/wake and HPC theta/delta ratio for REM/NREM), calculate the average stay probability.
- Identify the local minima, which correspond the centre of the transition zone.
- Identify the edges of the transition zone as a percentage of the minima (50%, in our case).

Step 3: identify true and aborted transitions (example of sleep to wake)

- Identify all times at which OB gamma power crosses the limit between the sleep state and the transition zone. If the next crossing of the transition zone limits is to the wake state, this is a ‘true transition’; if the next crossing is a return to the sleep state, this is an ‘aborted transition’.

Step 4: diffusion analysis

- Identify all true transitions
- Align OB gamma power of these transitions so that time 0 corresponds to the time at which the limit of the transition zone is crossed. This yields a set of trajectories across the transition zone. We suggest retaining 4 s of data after the crossing. Subtract the gamma power at time 0 and square the result to obtain the mean square displacement in time for each trajectory.
- Average the mean square displacement across all trajectories.
- Fit the resulting curve using the equation *MSD*(*t*) = 2*Dt*^*α*^.
- D and α can be interpreted as explained in the text.

### Box-Cox transformation

When comparing the strength of two correlations, linearity between the two is assumed, which is not necessarily true of biological data. One option is to use rank statistics such as the Spearman correlation coefficient; however, this method ignores the metric distance between values and therefore can stretch/compress values, depending on local density of points. We therefore preferred to transform data using the Box-Cox transformation. This is in fact a family of transformations that vary continuously depending on the variable λ and covers a large ensemble of transformations.

$$T(x,\lambda )=\frac{x^{\lambda }-1}{\lambda },if\;\lambda \neq 0$$

T(x,λ)=log(x),ifλ=0

Computing the Pearson correlation coefficient between the transformed variable and the parameter of interest thus allows us to find the value of λ, for which the correlation coefficient is maximal. This allows a fair comparison of the strength of correlation of two variables by taking into account their respective nonlinearities.

### ROC curves

To quantify the relationship between stimulus reactivity and OB gamma or heart rate, we used the ROC. We first classified stimulus responses into ‘sedated’ (i.e., below threshold) and ‘aroused’ (i.e., above threshold) based on the clearly bimodal distribution of response levels.

The ROC analysis quantifies the ability of an ideal observer to predict whether the animal’s response was ‘sedated’ or aroused based purely on the preceding OB gamma power.

This analysis presents the advantage over other evaluations of classifier accuracy of being insensitive to class distribution, i.e., the respective number of ‘sedated’ or ‘aroused’ stimulations, in our case, which can often be strongly skewed depending on the behaviour of the animal.

In our case, the observer discriminates the two response levels by placing a threshold (z) on the OB gamma power (γ) during the 3 s prior to stimulation below which the corresponding stimulation is classified as ‘sedated’ and above which it is classified as ‘aroused’. The performance of this procedure can be fully determined by two parameters:

α(z)=P(γ>Z|sedated)orfalsepositiverate

β(z)=P(γ>Z|aroused)ortruepositiverate

Plotting α and β for increasing values of z yields the ROC curve, and the area under this curve (ROC value) represents the probability that an ideal observer can discriminate between the two stimulus response levels based on preceding OB gamma power: it is equal to 0.5 if the spectral power carries no information about the behaviour and equals 1 if it is perfectly predictive.

## Supporting information

S1 Fig Stability of OB gamma power thresholds up to 2 mo. (A) Evolution of scoring overlap between sleep and wake states defined using thresholds defined on days 0 or on the day of recording as a function of time. (Error bars are s.e.m.: *n* = 14 mice with 3–15 recording sessions per mouse). (B) OB gamma power distributions and corresponding threshold from the same mouse over 1 mo (colour coded from first to last day). OB, olfactory bulb.
(TIF)

S2 Fig Application of OB gamma based sleep scoring to fluoxetine experiment. (A) Percentage of REM sleep is decreased after fluoxetine administration (15 mg/kg) compared to saline using novel OB-based sleep scoring method, as expected from previous results using movement-based scoring (*n* = 4, Friedman test χ^2^(3) = 4, *p* = 0.045). (B C) Overlap of EMG-based scoring with OB gamma scoring is equally high after saline (B) and fluoxetine (C) injection. Each column gives the percent of time from each state identified using EMG scoring (x label) that is classified as NREM (blue), REM (red), and wake (grey), respectively, using OB gamma scoring (*n* = 4). (D) Phase space of brain states showing the distribution of NREM (blue), REM (red), and wake (grey) after saline and fluoxetine administration. Corresponding histograms are shown along the relevant axis with automatically determined thresholds in red. (E) Heat map of point density averaged over all phase spaces for all mice (*n* = 4). Circles show the 95% boundaries of NREM, REM, and wake for each mouse either after saline (light colours) or fluoxetine (dark colours) injection. Histograms from all mice are shown along the relevant axis with automatically determined thresholds (*). For comparison, the data from each mouse are normalised by dividing the instantaneous power by the average NREM power to align all distributions to the peak of distribution of NREM activity. EMG, electromyography; NREM, non-REM; OB, olfactory bulb; REM, rapid eye movement.
(TIF)

S3 Fig OB gamma–based sleep scoring validation in four different mouse strains. (A) High-frequency spectra from C57Bl/6J Rj (*n* = 10), Gad2-IRES-Cre knock-in C57BL/6J Rj (*n* = 4), C3H/HeNRj (*n* = 2), and DBA/2Rj (*n* = 3) mice during wake, NREM, and REM sleep scored using movement-based scoring. The 50–70 Hz gamma band is shown in pink. (B) Difference between wake and sleep spectra in the same four mouse lines. Note that all four lines display a clear peak in the gamma activity band (pink bar); this power peaks at a slightly lower frequency in C3H and DBA mice. The insets show the same sleep/wake difference of spectra z-scored and compared for each of the three lines to the C57Bl/6J Rj line. (Error bars are s.e.m.). (C) Overlap of EMG-based scoring with OB gamma scoring. Each column gives the percentage of time from each state identified using EMG scoring (x label) that is classified as NREM (blue), REM (red), and wake (grey), respectively, using OB gamma scoring. (D) Example data set for all three mouse strains showing HPC low-frequency spectrogram with theta/delta power ratio below and OB high-frequency spectrogram with gamma power below. The hypnogram is shown at the bottom. The relevant frequency bands are outlined by a dotted grey line. (E) Phase space of brain states showing the distribution of NREM (blue), REM (red), and wake (grey) for all three mouse strains. Corresponding histograms are shown along the relevant axis, with automatically determined thresholds in red. EMG, electromyography; HPC, hippocampus; NREM, non-REM; OB, olfactory bulb; REM, rapid eye movement.
(TIF)

S4 Fig Piriform cortex gamma tracks sleep and wake. (A) Phase space of brain states showing the distribution of NREM (blue), REM (red), and wake (grey) from an example mouse. Corresponding histograms are shown along the relevant axis, with automatically determined thresholds in red. (B) Overlap of EMG scoring with piriform cortex gamma scoring. Each column gives the percent of time from each state identified using manual scoring (x label) that is classified as NREM (blue), REM (red), and wake (grey), respectively, using piriform cortex gamma scoring (*n* = 2). EMG, electromyography; NREM, non-REM, REM, rapid eye movement.
(TIF)

S5 Fig Dependency of gamma power in HPC on recording layer. (A) High-frequency spectra from HPC as in Fig 1B but restricted to pyramidal layer recordings only. This shows the increase in gamma activity during wake and REM states relative to NREM state. (B) Activity recorded with a silicon probe in the HPC triggered on the trough of theta oscillation (top) and sharp wave ripple events (bottom). Averaged event-triggered LFPs and the derived CSDs are shown, illustrating the expected sink/source pairs. (C) High-frequency spectra from same HPC recording layers as shown in B. Note that the gamma power difference between REM/wake and NREM is strongest in the pyramidal layer and changes with recording depth. CSD, current source density; HPC, hippocampus; LFP, local field potential; NREM, non-REM; REM, rapid eye movement.
(TIFF)

S1 Movie Example data from sleep session. Left: Phase space of brain states showing the distribution of NREM (blue), REM (red), and wake (grey). The moving line shows the position of current data points. Right: Top shows data from ECG showing thoracic muscular activity (ECG), PFC LFP and EEG (dark), HPC, and OB recordings during period shown by black line on spectrograms of OB and HPC at bottom. PFC amplitude has been increased by 25% to increase visibility. Superimposed on spectrograms are the OB gamma power and HPC theta/delta ratio used for scoring with the relevant thresholds shown in red. EEG, electroencephalography; HPC, hippocampus; LFP, local field potential; NREM, non-REM; OB, olfactory bulb; PFC, prefrontal cortex; REM, rapid eye movement.
(AVI)

## Abbreviations

AP: anteroposterior
BIS: bispectral index
CS+: conditioned stimulus associated with shock
CS−: conditioned stimulus not associated with shock
DV: dorsoventral
EEG: electroencephalography
EMG: electromyography
epl: external plexiform layer
gr: glomerular layer
HPC: hippocampus
LDA: linear discriminant analysis
LFP: local field potential
ML: mediolateral
ns: not significant
OB: olfactory bulb
ROC: receiver operator characteristic
SVM: support vector machine
