# Supplementary figures and images for: Harnessing olfactory bulb oscillations to perform fully brain-based sleep-scoring and real-time monitoring of anaesthesia depth
Source: PLoS Biol. 2018 Nov 8;16(11):e2005458. doi: 10.1371/journal.pbio.2005458 (PMC6224033; doi:10.1371/journal.pbio.2005458)

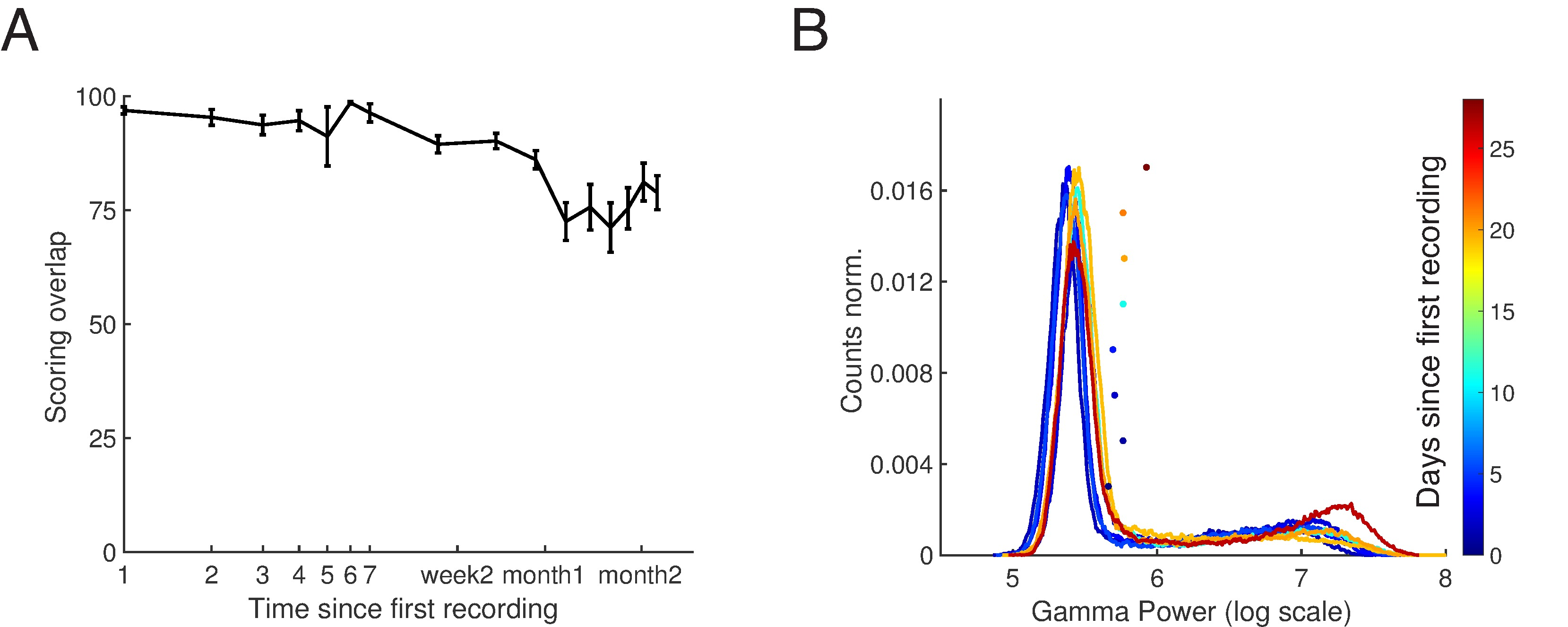

Supplement: S1 Fig — (A) Evolution of scoring overlap between sleep and wake states defined using thresholds defined on days 0 or on the day of recording as a function of time. (Error bars are s.e.m.: n = 14 mice with 3–15 recording sessions per mouse). (B) OB gamma power distributions and corresponding threshold from the same mouse over 1 mo (colour coded from first to last day). OB, olfactory bulb. (TIF) [file pbio.2005458.s001.tif]

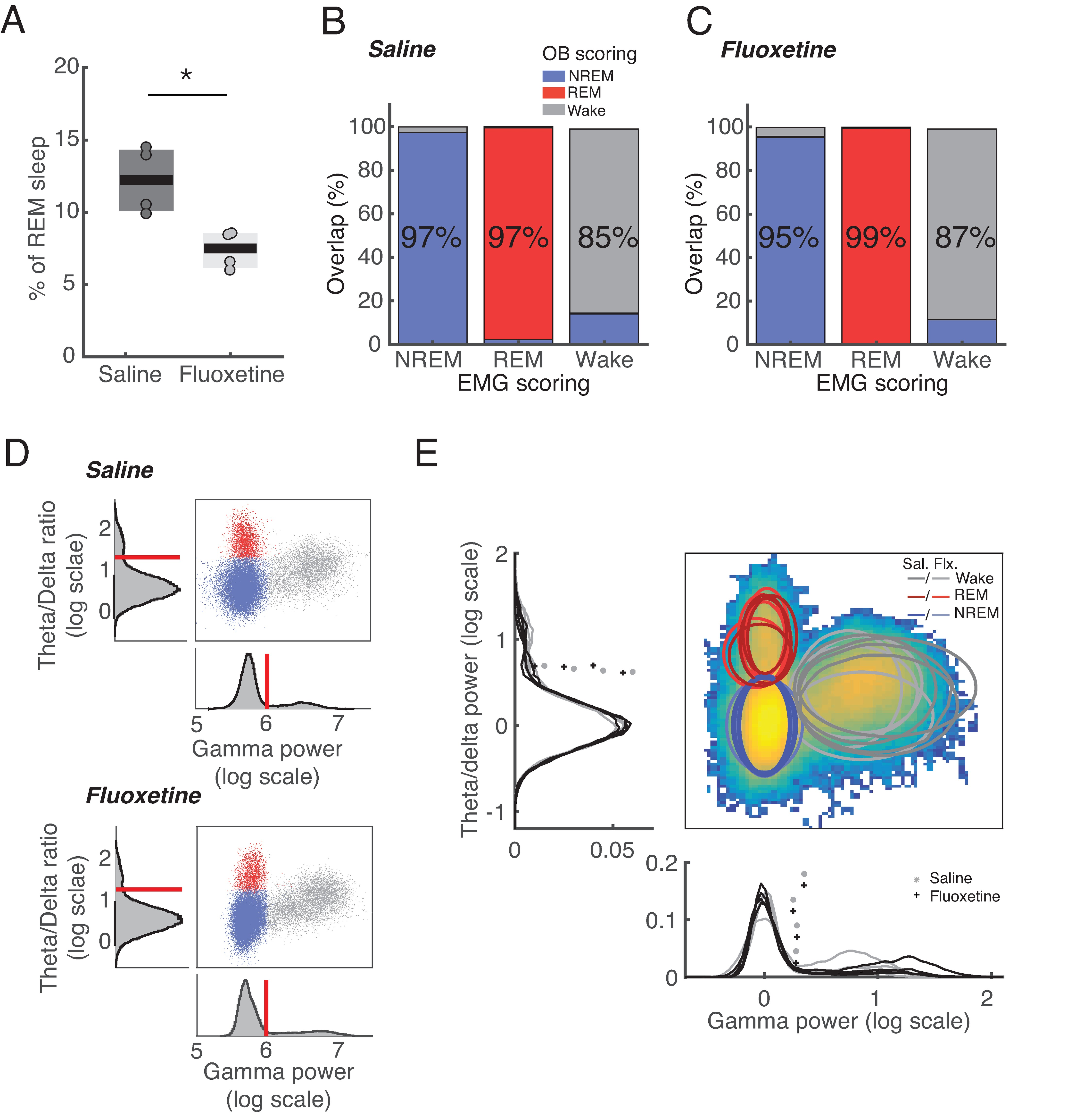

Supplement: S2 Fig — (A) Percentage of REM sleep is decreased after fluoxetine administration (15 mg/kg) compared to saline using novel OB-based sleep scoring method, as expected from previous results using movement-based scoring (n = 4, Friedman test χ2(3) = 4, p = 0.045). (B C) Overlap of EMG-based scoring with OB gamma scoring is equally high after saline (B) and fluoxetine (C) injection. Each column gives the percent of time from each state identified using EMG scoring (x label) that is classified as NREM (blue), REM (red), and wake (grey), respectively, using OB gamma scoring (n = 4). (D) Phase space of brain states showing the distribution of NREM (blue), REM (red), and wake (grey) after saline and fluoxetine administration. Corresponding histograms are shown along the relevant axis with automatically determined thresholds in red. (E) Heat map of point density averaged over all phase spaces for all mice (n = 4). Circles show the 95% boundaries of NREM, REM, and wake for each mouse either after saline (light colours) or fluoxetine (dark colours) injection. Histograms from all mice are shown along the relevant axis with automatically determined thresholds (*). For comparison, the data from each mouse are normalised by dividing the instantaneous power by the average NREM power to align all distributions to the peak of distribution of NREM activity. EMG, electromyography; NREM, non-REM; OB, olfactory bulb; REM, rapid eye movement. (TIF) [file pbio.2005458.s002.tif]

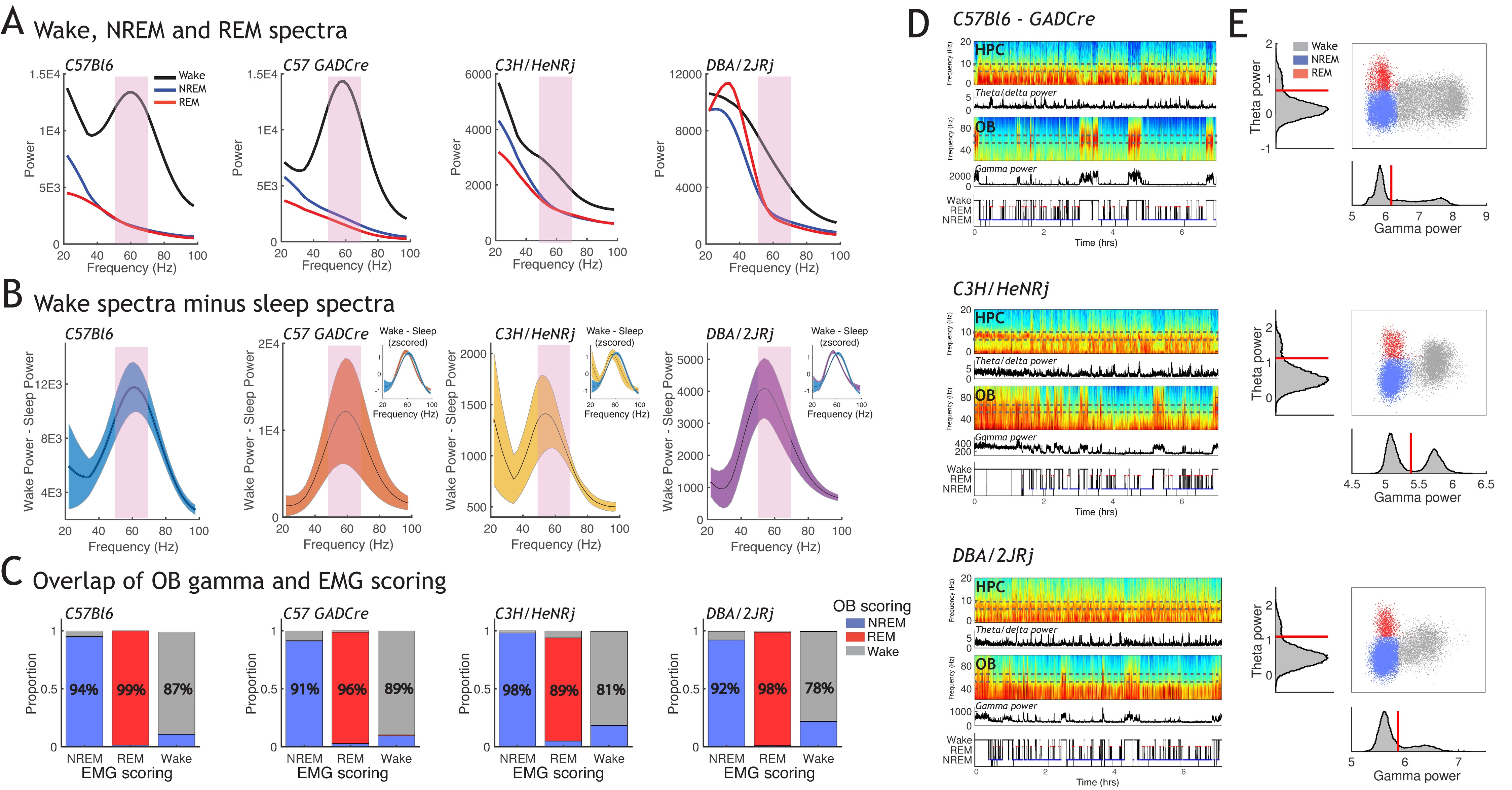

Supplement: S3 Fig — (A) High-frequency spectra from C57Bl/6J Rj (n = 10), Gad2-IRES-Cre knock-in C57BL/6J Rj (n = 4), C3H/HeNRj (n = 2), and DBA/2Rj (n = 3) mice during wake, NREM, and REM sleep scored using movement-based scoring. The 50–70 Hz gamma band is shown in pink. (B) Difference between wake and sleep spectra in the same four mouse lines. Note that all four lines display a clear peak in the gamma activity band (pink bar); this power peaks at a slightly lower frequency in C3H and DBA mice. The insets show the same sleep/wake difference of spectra z-scored and compared for each of the three lines to the C57Bl/6J Rj line. (Error bars are s.e.m.). (C) Overlap of EMG-based scoring with OB gamma scoring. Each column gives the percentage of time from each state identified using EMG scoring (x label) that is classified as NREM (blue), REM (red), and wake (grey), respectively, using OB gamma scoring. (D) Example data set for all three mouse strains showing HPC low-frequency spectrogram with theta/delta power ratio below and OB high-frequency spectrogram with gamma power below. The hypnogram is shown at the bottom. The relevant frequency bands are outlined by a dotted grey line. (E) Phase space of brain states showing the distribution of NREM (blue), REM (red), and wake (grey) for all three mouse strains. Corresponding histograms are shown along the relevant axis, with automatically determined thresholds in red. EMG, electromyography; HPC, hippocampus; NREM, non-REM; OB, olfactory bulb; REM, rapid eye movement. (TIF) [file pbio.2005458.s003.tif]

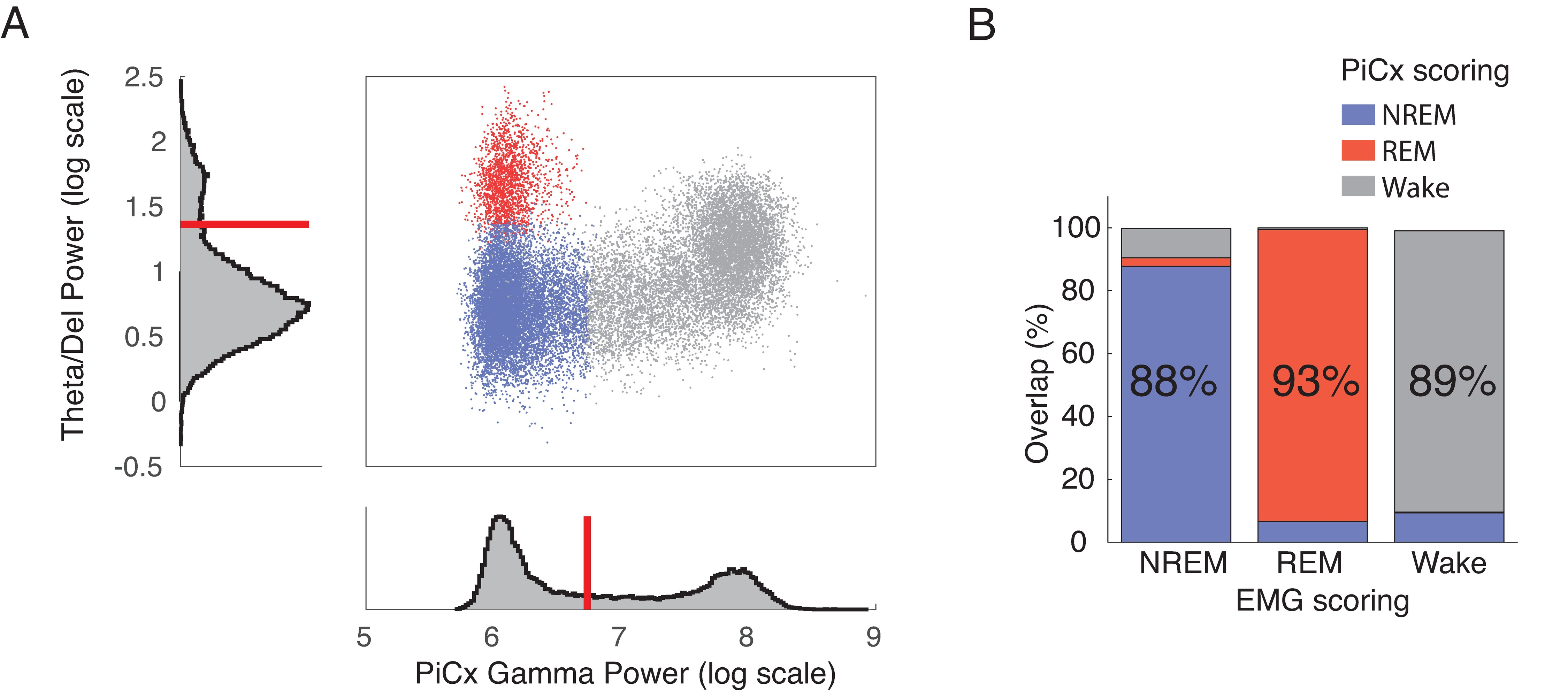

Supplement: S4 Fig — (A) Phase space of brain states showing the distribution of NREM (blue), REM (red), and wake (grey) from an example mouse. Corresponding histograms are shown along the relevant axis, with automatically determined thresholds in red. (B) Overlap of EMG scoring with piriform cortex gamma scoring. Each column gives the percent of time from each state identified using manual scoring (x label) that is classified as NREM (blue), REM (red), and wake (grey), respectively, using piriform cortex gamma scoring (n = 2). EMG, electromyography; NREM, non-REM, REM, rapid eye movement. (TIF) [file pbio.2005458.s004.tif]

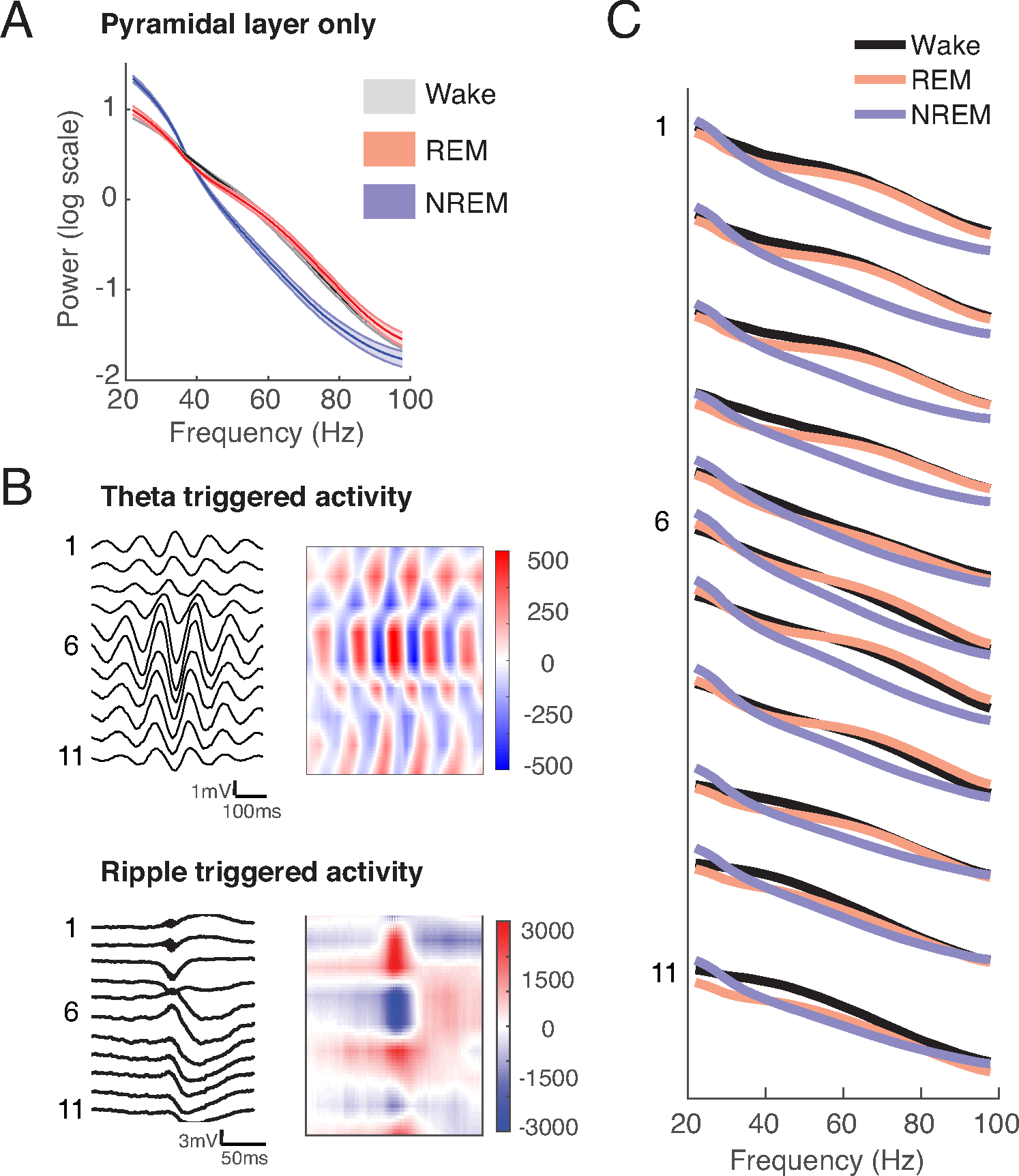

Supplement: S5 Fig — (A) High-frequency spectra from HPC as in Fig 1B but restricted to pyramidal layer recordings only. This shows the increase in gamma activity during wake and REM states relative to NREM state. (B) Activity recorded with a silicon probe in the HPC triggered on the trough of theta oscillation (top) and sharp wave ripple events (bottom). Averaged event-triggered LFPs and the derived CSDs are shown, illustrating the expected sink/source pairs. (C) High-frequency spectra from same HPC recording layers as shown in B. Note that the gamma power difference between REM/wake and NREM is strongest in the pyramidal layer and changes with recording depth. CSD, current source density; HPC, hippocampus; LFP, local field potential; NREM, non-REM; REM, rapid eye movement. (TIFF) [file pbio.2005458.s005.tiff]
